# Left ventricular segmentation from MRI datasets with edge modelling conditional random fields

**DOI:** 10.1186/1471-2342-13-24

**Published:** 2013-07-31

**Authors:** Janto F Dreijer, Ben M Herbst, Johan A du Preez

**Affiliations:** 1 Department of Applied Mathematics, Stellenbosch University, Stellenbosch, South Africa; 2 Department of Electrical and Electronic Engineering, Stellenbosch University, Stellenbosch, South Africa

## Abstract

**Background:**

This paper considers automatic segmentation of the left cardiac ventricle in short axis magnetic resonance images. Various aspects, such as the presence of papillary muscles near the endocardium border, makes simple threshold based segmentation difficult.

**Methods:**

The endo- and epicardium are modelled as two series of radii which are inter-related using features describing shape and motion. Image features are derived from edge information from human annotated images. The features are combined within a discriminatively trained Conditional Random Field (CRF). Loopy belief propagation is used to infer segmentations when an unsegmented video sequence is given. Powell’s method is applied to find CRF parameters by minimizing the difference between ground truth annotations and the inferred contours. We also describe how the endocardium centre points are calculated from a single human-provided centre point in the first frame, through minimization of frame alignment error.

**Results:**

We present and analyse the results of segmentation. The algorithm exhibits robustness against inclusion of the papillary muscles by integrating shape and motion information. Possible future improvements are identified.

**Conclusions:**

The presented model integrates shape and motion information to segment the inner and outer contours in the presence of papillary muscles. On the Sunnybrook dataset we find an average Dice metric of 0.91±0.02 and 0.93±0.02 for the inner and outer segmentations, respectively. Particularly problematic are patients with hypertrophy where the blood pool disappears from view at end-systole.

## Background

**Figure 1:**
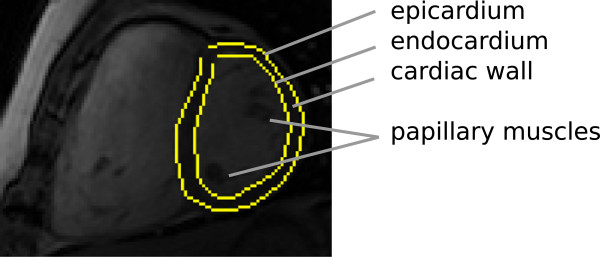
**MRI short axis view.** MRI short axis view of ventricles with human annotated (inner and outer) contours shown in yellow. Surrounding tissue is omitted for illustrative purposes.

Properties of the cardiac left ventricle, such as volume, ejection fraction and wall thickness are important indicators for the diagnosis of many heart-related problems. Many of these are conveniently extracted from Magnetic Resonance Imaging (MRI). Calculating these properties requires accurate annotation of the left ventricle to isolate it from its surrounding structure. Figure 1 illustrates the cardiac structure and human annotated inner and outer contours of the left ventricle in an MRI image.

Manual annotation is a tedious process and lacks consistency between human annotators [1,2] and even between separate annotations by the same annotator.

Various properties of magnetic resonance images, such as intensity inhomogeneities, z-shift due to respiration and imaging acquisition artifacts, can make segmentation difficult. One of the most severe problems arises from judging to what extent the papillary muscles influence and, possibly, obscure the endocardium border. For research on the effects that discrepancies in annotations of the papillary muscles can have on the calculation of left ventricle function and mass see e.g. [2-4]. In this work we primarily focus on mitigating the effect of the papillary muscles.

**Figure 2:**
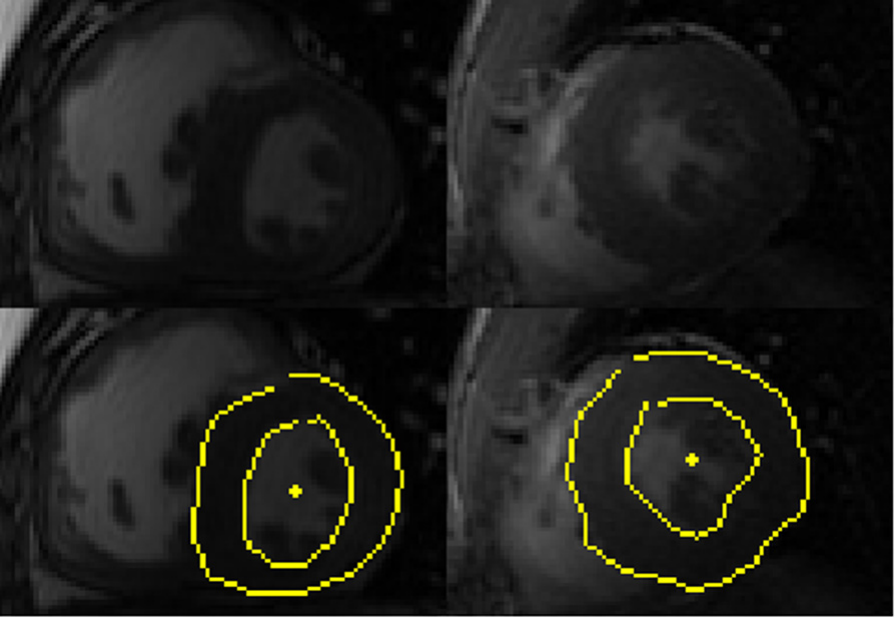
**Papillary muscles obscuring edge.** Presence of papillary muscles obscure the edge of the inner contour due to its similar intensity to the cardiac wall. Human annotated inner and outer contours are shown in yellow. Surrounding tissue is cropped for clarity.

The examples in Figure 2 illustrate the presence of papillary muscles close to the endocardium border and a human annotator’s segmentation. When modelling the structural properties of the ventricle wall, it is often desirable to include these muscles inside the inner contour. From an inspection of the human annotations, it is clear that the presence of the endocardium border is inferred from information other than only a strong intensity gradient, possibly from prior knowledge of the motion and shape of the ventricle. Such considerations are difficult to integrate into a simple intensity threshold-based techniques of a single image, in particular when there is little difference between the intensity of the endocardium and surrounding structure.

A number of automated techniques have been developed for the segmentation of the left ventricle from its surrounding structure (see e.g. [5,6]). For a review of snakes and deformable models in medical image analysis see e.g. [7]. We will briefly discuss the most popular techniques.

Active Shape Models (ASMs, [8]) track the edges in a video sequence by modelling the contour shape using methods such as Principal Component Analysis. This is often combined with a Kalman filter to incorporate temporal smoothing in an online tracking framework. Typically only past information is used and future images ignored, often with adverse consequences if the object being tracked disappears from view or becomes very small.

Andreopoulos and Tsotsos [9] fit a 3D Active Appearance Model (AAM) and investigate a hierarchical “2D + time” ASM that integrates temporal constraints by using the third dimension for time instead of space.

Generative models such as Markov Random Fields (MRF) are popular in pixel labelling and de-noising problems [10]. Modelling the image probability can, however, lead to intractable models with complex dependencies between local features. This can lead to reduced performance if oversimplified [11]. Careful manual design of the probability distributions is therefore often necessary.

Most image segmentation applications of MRFs also model the texture within a region and are constructed to favour spatially smooth regions. We refer to these models as Surface MRFs. Surface MRFs are often used to isolate homogeneous objects from their backgrounds. The left ventricle, however, contains papillary muscles (see Figures 1 and 2), rendering this approach less effective. As surface MRFs do not model the edge explicitly, they do not directly encode any shape information. There have been attempts to unify models of the edge and surface: specifically, Huang et al. [12] propose coupling surface MRFs with a hidden state representing a deformable contour.

Cordero-Grande et al. [13] construct an MRF model of the inner and outer contours, using a purely generative model. They use the Gibbs sampling algorithm to extract segmentations and parameters.

Also of interest is the approach by Jolly [14], in which the segmentation problem is set in log-polar space where the least cost path in a single frame (calculated by dynamic programming) is defined as the desired contour. A cost function, which they relate to an initial labelling of blood pool pixels, is required to determine the correct contour. This is similar to our approach in that if we limit our model to a single frame (i.e. remove temporal linkage) belief propagation also reduces to estimating the least cost path via dynamic programming.

In summary, a number of methods are available, with the main challenge being integration of temporal and spatial constraints.

In an earlier study, Dreijer et al. [15] investigated modelling the endocardium edge using a semi-conditional MRF with mostly heuristically chosen features. Although this approach showed promise, practical experiments indicated a tendency to “snap” onto the epicardium, partly due to the epicardium’s stronger edge with respect to surrounding tissue. In the present study, the outer contour is explicitly included in the model, establishing a statistical dependency between the two contours. We also train discriminative feature functions from human annotated images as opposed to the heuristically chosen functions used previously.

## Methods

### Conditional random field

A Conditional Random Field (CRF, [16]) models the conditional probability of a set of unobserved/latent variables, ***y***, given a set of observations ***x***, i.e. *P*(***y***|***x***). This is different from a generative Markov random field which models the probability distribution of the observed variables, given different configuration of the latent variables, i.e. *P*(***x***|***y***).

A CRF represents the modelling probability as a product of local potential functions, defined over subsets of the latent variables,

(1) $$P\left(y|x\right)=\frac{1}{Z}\underset{c\subset y}{\prod }\psi_{c}\left(y_{c},x\right)$$

where the normalizing partition function $Z=\sum_{y}\prod_{c\subset y}\psi_{c}\left(y_{c},x\right)$ sums over all possible configurations of ***y***.

The main advantage of a CRF is its discriminative nature, i.e. it does not require a detailed model of the observed information, instead, computational resources are dedicated to describing the latent variables.

### Problem formulation

We refer to the left ventricle endocardium as the inner contour and the left ventricle epicardium together with the right ventricle’s endocardium (bordering the septum) as the outer contour, see Figure 1.

We are primarily interested in segmenting a video sequence of *T* grayscale images, *I*(0),…,*I*(*T* − 1), that is synchronised with a single cardiac cycle so that the first image *I*(0) is before systole (contraction) and the last image, *I*(*T* − 1), after diastole (relaxation). End-systole (maximum contraction) thus occurs in the middle of the video sequence at approximately *I*(*T*/2). We refer to the grayscale value of a pixel within a single image at the *x*,*y* coordinate ***p*** as *I*(*t*,***p***).

**Figure 3:**
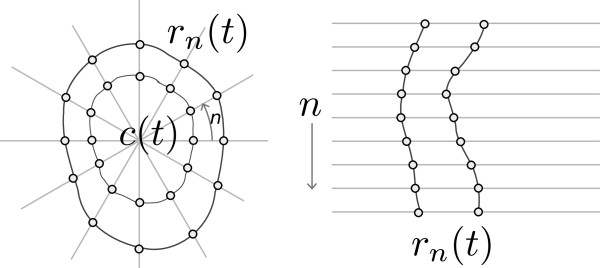
**Cartesian and polar coordinates.** A representation of coordinates on the inner and outer contours in Cartesian and polar coordinates.

Assuming an annular shape, an inner or outer contour in a frame at time *t*, can be represented by a sequence of *N* radii, *r*_*n*_(*t*), at uniformly spaced angles, *n* = 0,…,*N* − 1 around a shared centre point, ***c***(*t*). Figure 3 illustrates coordinates on the inner and outer contours using a small number of radii. In our implementation we use *N*=128 angular directions.

We prefer to work in the discretised log-space of the radii, i.e.

(2) $$\rho_{n}(t)=\left\lfloor M\cdot r_{\text{init}}\cdot \log r_{n}(t)\right\rfloor$$

where ⌊*x*⌋ is the floor function of *x* and *r*_init_=50 is experimentally chosen such that, for most segmentations in the training set, *ρ*_*n*_(*t*)≈*M*/2 at end-diastole. The radius is discretised as *ρ*_*n*_∈{0,…,*M* − 1} where *M*=256 provides a resolution sufficient for human segmentation of the transformed image. One advantage of the log-space is that it provides a better spatial resolution at smaller radii.

**Figure 4:**
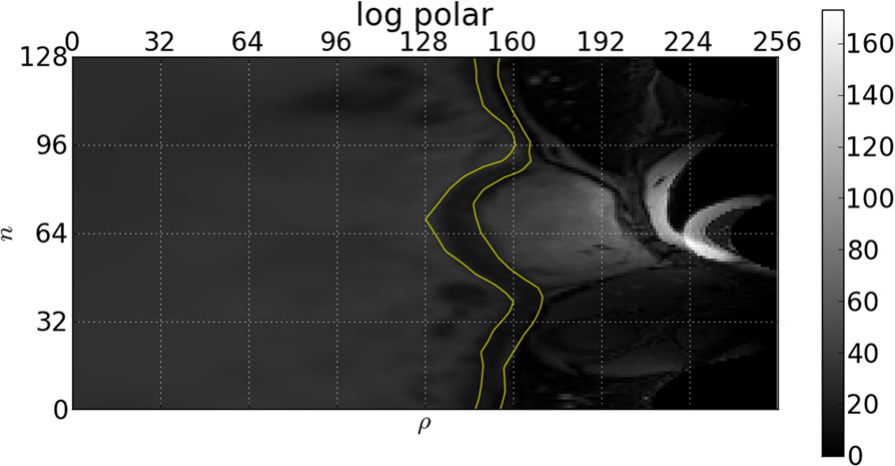
**Log-polar transform.** Log-polar transform with human annotated inner and outer contours in yellow. The centre point is chosen near the middle of the blood pool. Ground truth inner and outer contours are indicated with solid yellow lines.

Figure 4 illustrates the log-polar transformed image, *D*(*t*), of a single image, *I*(*t*). We denote image values in a radial direction (i.e. a row in the log-polar image), *n*, as the vector ***d***_*n*_(*t*). The grayscale value of a pixel in the log-polar space is then referred to as ***d***_*n*_(*t*,*ρ*).

The two segmentation contours in a single frame are thus fully represented as two vectors of log-radii,

(3) $$\begin{array}{ll} \rho^{\text{in}}(t)= & \left\{\rho_{0}^{\text{in}}(t),\ldots ,\rho_{N\;-\;1}^{\text{in}}(t)\right\}\; \end{array}$$

(4) $$\begin{array}{ll} \rho^{\text{out}}(t)= & \left\{\rho_{0}^{\text{out}}(t),\ldots ,\rho_{N\;-\;1}^{\text{out}}(t)\right\},\; \end{array}$$

around the centre point ***c***(*t*). The segmentation of a video sequence of frames is represented by ***ρ***={***ρ***^in^,***ρ***^out^} around a series of centre points ***c***={***c***(*t*)}_*t*=0,…,*T* − 1_ where

(5) $$\begin{array}{ll} \rho^{\text{in}}= & \left\{\rho^{\text{in}}(0),\ldots ,\rho^{\text{in}}(T\;-\;1)\right\}\; \end{array}$$

(6) $$\begin{array}{ll} \rho^{\text{out}}= & \left\{\rho^{\text{out}}(0),\ldots ,\rho^{\text{out}}(T\;-\;1)\right\}.\; \end{array}$$

Note that the position of the centre point is different for each frame. Partly due to the non-symmetrical contraction of the heart, the ventricle centre point can undergo significant translation.

Our segmentation process first determines a series of centre points, after which relative radial values are inferred. In the next section we turn our attention to finding suitable centre points.

### Centre point estimation

Our CRF model requires the centre points to be placed near the middle of the inner contour allowing the model to restrict the spatial and temporal variability of the radii. As a semi-automatic clinical tool, a human operator could annotate the centre point in every frame of the video sequence. In our work towards a fully-automatic technique we describe a semi-automated procedure that requires only annotating the centre point in one frame, after which the other centre points are estimated. Fully-automatic techniques are especially valuable when analysing significantly large datasets.

A number of heuristic techniques (e.g. [5]) are available for estimating the centre points. Most of these perform adequately when the papillary muscles are absent and there is high contrast between the blood pool and cardiac wall. This is generally the case for the first few frames, but not at the end of systole when the ventricle is at its smallest.

The procedure described below requires the user to provide the centre point ***c***(0) of the first frame when the left ventricle blood pool is clearly visible and the papillary muscles minimally obstruct the inner contour. The robustness against variations in ***c***(0) is discussed in Section “Sensitivity to initial centre point placement”. With an appropriate user interface the annotation of the center point in the first frame of all spatial slices would only take the user a few seconds.

Since each video sequence from our dataset contains a single heart beat, and due to its periodic nature, we assume the last frame has the same centre point as the first, i.e. ***c***(*T* − 1)=***c***(0). This is not a severe restriction since the algorithm is robust against variations in ***c***(*t*), as demonstrated in Section “Sensitivity to initial centre point placement”.

To find centre points in the intermediate frames, ***c***(1),…,***c***(*T* − 2), we minimize a weighted between-frame alignment error,

(7) $$\;\text{error}\left(c\right)\;=\;\;\overset{T-1}{\underset{t=1}{\sum }}\underset{p}{\sum }w^{c(t)}\left(p\right)\cdot {\left(I^{c(t)}\left(t,p\right)\;-\;I^{c\left(t-1\right)}\left(t\;-\;1,p\right)\right)}^{2},$$

where *I*^***c***(*t*)^(*t*) is the image *I*(*t*) centred at ***c***(*t*) such that *I*^*c*(*t*)^(*t*,***p***)=*I*(*t*,***p***−***c***(*t*)) and zero when indexed out of bounds, ***p*** is, again, the *x*,*y* coordinates and *I*(*t*) the frame at time *t* before a log-polar transform is applied.

The weight $w^{c(t)}\left(p\right)=e^{\left(-{∥c(t)-p∥}^{2}/\sigma^{2}\right)}$ locally enhances the error around the frame’s centre point. The width, *σ*, is experimentally chosen from a training dataset so that the inferred centre points closely resemble the mean of the ground truth inner contours. For computational efficiency we only calculate values within a 2*σ* radius of ***c***(*t*) as the contribution becomes negligible further away.

Note that (7) is a nonlinear function of the sequence of centre points, but is efficiently solved using an optimization strategy such as dynamic programming. In addition, in order to reduce computational cost a beam search is implemented, effectively assuming the centre point translates less than three pixels between frames. Backtracking is initiated at the centre of the last frame ***c***(*T* − 1). In order to constrain ***c***(0) to the value provided by the user, during dynamic programming, the cumulative cost function for the first frame is set to zero at that value and 1 otherwise.

We pose the centre point estimation problem as one that can be solved within the same belief propagation framework as our radial inference. However, since this is independent of the radial inference fast heuristic methods could be considered in the future.

### The CRF model

Representing a temporal sequences of log-polar images as *D*={*D*(*t*)}_*t*=0,…,*T*−1_, and assuming appropriately trained parameters ***θ*** we model the conditional probability *P*(***ρ***|***θ***,*D*) of a segmentation ***ρ***. This is done through a log-linear CRF,

(8) $$P\left(\rho |\theta ,D\right)=\frac{1}{Z\left(\theta ,D\right)}\exp\left(-E\left(\rho |\theta ,D\right)\right).$$

The energy *E*(***ρ***|***θ***,*D*) is defined as the weighted sum of local feature functions ***f*** defined over all cliques q∈Q in its graphical model

(9) $$E\left(\rho |\theta ,D\right)=\underset{q\in Q}{\sum }\theta_{q}\;f_{q}\left(\rho_{q},D\right).$$

In this formulation, smaller energies indicate better segmentations, while bad segmentations are penalized with larger energies.

The partition function

(10) $$Z\left(\theta ,D\right)=\underset{\rho }{\sum }\exp\left(-E\left(\rho |\theta ,D\right)\right)$$

sums over all possible configurations of ***ρ***, normalizing the exponentiated energy into a probability.

Note that there are no significant theoretical restrictions to the feature functions in a random field. Any further restrictions and choices of feature functions are design choices made to improve performance.

### Feature functions

We restrict ourselves to positive feature functions ***f*** where small values are more desirable (i.e. smaller values should correlate with “better” segmentations). Positive parameters, ***θ***, determine the relative weights of the feature functions. In order to minimize the clique size, we also restrict ourselves to features that couple at most two radial values in space and time, as computational cost grows exponentially with increasing clique size.

**Figure 5:**
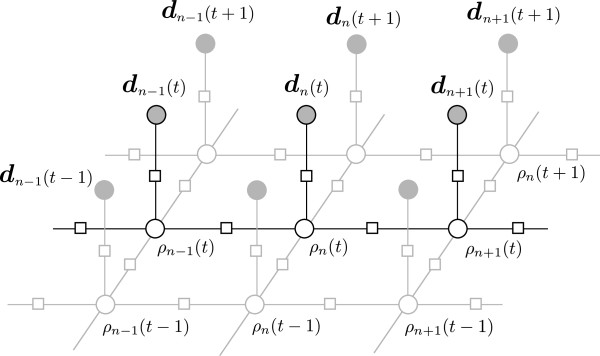
**Partial factor graph.** A partial factor graph of the temporal and spatial relationships between radius variables, ***ρ***, in a single contour and rows in the log-polar transformed image, ***d***_*n*_(*t*). Factor labels and some variable labels are omitted for clarity.

A partial factor graph [17] in Figure 5 illustrates the temporal and spatial relationships between radius variables, ***ρ***, in a single contour and rows in the log-polar transformed image, ***d***_*n*_(*t*). The spatially circular nature (i.e. there is a feature connecting *ρ*_0_(*t*) and *ρ*_*N*−1_(*t*)) and the relationships between the inner and outer contours are omitted for clarity.

We derive the feature functions from discriminative properties of human annotated images as described below. Selection of the relative weights, ***θ***, is discussed in Section “CRF parameter estimation”.

#### Feature function based on edge classifiers

For a log-polar frame (such as in Figure 4) at time *t*, consider a window of height 1 and width $w=\frac{M}{4}=64$ around a radius *ρ* in the radial direction *n*. This window is equivalent to a circular sector in the original image before the log-polar transform is applied. We refer to the pixel values in this window as the vector $v_{\rho }=d_{n}\left(t,\left[\rho -\frac{w}{2},\ldots ,\rho +\frac{w}{2}\right]\right)$. A feature vector *κ*(***v***) is derived from the window and is described below.

We train an artificial neural network, with two nodes in a hidden layer, to model the presence of the cardiac edge. A window extracted from the training set is considered to contain an edge if the centre of the window is no more than 2 radial distances away from the ground truth edge, otherwise it is considered a “non-edge” training example.

The feature vector, *κ*(***v***), consists of the concatenation of four expressions of the gradient in the radial direction, $\frac{\partial v}{\partial \rho }$, that we suspect the classifier might find useful in discriminating between edges and non-edges,

(11) $$\kappa \left(v\right)=\left(\frac{\partial v}{\partial \rho },\left|\frac{\partial v}{\partial \rho }\right|,\text{sign}\left(\frac{\partial v}{\partial \rho }\right),\left[\left|\frac{\partial v}{\partial \rho }\right|>\epsilon \right]\right).$$

The expression $\left[\left|\frac{\partial v}{\partial \rho }\right|>\epsilon \right]$ is a binary value indicating the presence of a gradient.

From the short-axis view in Figure 1, it is worth noting that the gradient’s sign on the left and right sides of the outer contour’s edge differ, due to the intensity of the right ventricle’s blood pool. We therefore train eight classifiers for different parts of the contour, i.e. instead of training a single classifier over all angular directions (*n*=0..*N*−1) we treat groups of angles separately (*n*=0,…,15, *n*=16,…,31 etc.) and thus train direction dependent classifiers. This allows the classifiers to exploit features that it might find relevant in that direction.

We repeat the process for the classifiers of the inner contour’s edges as the endocardium’s edge behaviour also differs between the left and right sides of the ventricle due to the presence of the papillary muscles on mostly one side (see Figure 4).

Heiberg [6] identifies the edges of the inner and outer contours as two classes: Concordant, where edge gradients have a similar sign, and Discordant, areas where the edge gradient sign differ. Accordingly, he explicitly includes the gradient sign for the inner contour and ignores the sign for the outer. Because our edge model is trained on annotated ground truth images, our classifier automatically differentiates between the signs when they are relevant to edge detection.

To fit within the framework of energy minimization, the response of the neural network to an image is transformed into a cost by subtracting its output value from one. The minimum cost in the radial direction is subtracted to avoid negative feature values and is normalized by the sum.

We construct these networks for the inner and outer contour edges and so derive the features $f^{\text{in}}\left(\rho_{n}^{\text{in}}(t),d_{n}(t)\right)$ and $f^{\text{out}}\left(\rho_{n}^{\text{out}}(t),d_{n}(t)\right)$.

It should be noted that the typical neural network training techniques attempt to minimize a classification error, however we are not so much interested in the classification as using the output to penalise non-edges while at the same time minimally penalising edges. Effectively, we would prefer a classifier that rejects very few edges. To mitigate unintended penalisation, the outputs of the constructed features are “smoothed” by applying a small minimum-filter (erosion), in the angular direction. This will cause an area to have a high feature response (i.e. penalised) only if its neighbouring areas (in the angular direction) also have high responses.

**Figure 6:**
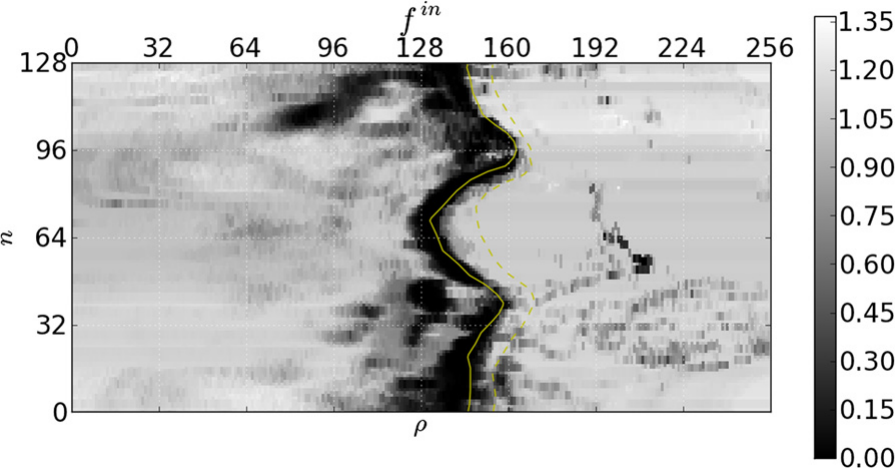
**Inner edge feature function.** Response of the feature function for the inner edges to the image in Figure 5. The ground truth inner contour is indicated with a solid yellow line and the outer with a broken line.

**Figure 7:**
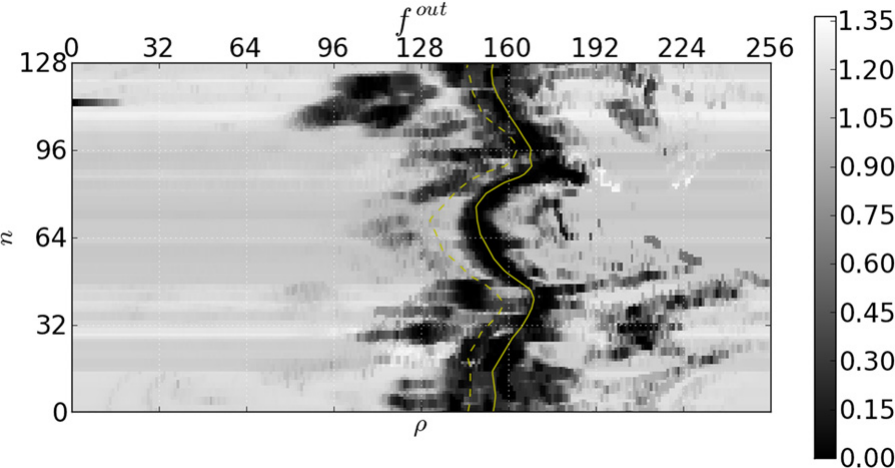
**Outer edge feature function.** Response of the feature function for the outer edges to the image in Figure 5. The ground truth outer contour is indicated with a solid yellow line and the inner with a broken line.

Figures 6 and 7, respectively, show the resulting inner and outer features’ responses to the frame in Figure 4. Note that the response is relatively low in the area of the ground truth. Recall that more desirable segmentations have smaller feature values. It should be emphasized that these response images have been obtained without taking into account any temporal behaviour or continuity requirements. We now investigate how to incorporate these properties into our model.

#### Spatial and temporal feature functions

Figure 4 shows that there are strong gradients at some non-contour positions and relatively weak gradients at some contour positions, especially when papillary muscles are close to the endocardium border, resulting in the feature responses of Figures 6 and 7. This is undesirable as inference from these features alone would cause incorrect segmentations in these areas.

We now proceed to introduce spatial continuity, as well as temporal information directly into the model by introducing the following feature functions,

(12) $$\begin{array}{ll} f_{r}\left(\rho_{n}(t),\rho_{n-1}(t)\right)= & {\left(\frac{\rho_{n}(t)-\rho_{n-1}(t)}{M}\right)}^{2},\; \end{array}$$

(13) $$\begin{array}{ll} f_{t}\left(\rho_{n}(t),\rho_{n}\left(t-1\right)\right)= & {\left(\frac{\rho_{n}(t)-\rho_{n}\left(t-1\right)}{M}\right)}^{2},\; \end{array}$$

where *M* is again the discretisation value, used here to scale the feature values to the same order of magnitude as the features described previously. From direct inspection, few contours violate the properties |*ρ*_*n*_(*t*)−*ρ*_*n*_(*t*−1)|≤25 and |*ρ*_*n*_(*t*)−*ρ*_*n*−1_(*t*)|≤2. This can be exploited during inference by applying a beam search and thereby significantly reducing the search space.

We also detect and penalize contour growth during systole and shrinkage during diastole by assuming end-systole (maximum contraction) is reached at time *t*_*ES*_,

(14) $$\;f_{t}^{\prime }\left(\rho_{n}(t),\rho_{n}\left(t-1\right)\right)=\left\{\begin{array}{ll} \left[\rho_{n}\left(t-1\right)<\rho_{n}(t)\right]\; & \text{if}\;t<t_{\text{ES}}\\ \left[\rho_{n}(t)<\rho_{n}\left(t-1\right)\right]\; & \text{otherwise.} \end{array}\right)$$

For simplicity, we have chosen a fixed *t*_*ES*_=8 from inspection of the training annotations. The correct selection of *t*_*ES*_ is sensitive to patient pathology. A more robust choice might depend on detecting when the optical flow in the images are suspended and reversed. A full discussion is, however, beyond the scope of this article. Again, these features are constructed separately for both the inner and outer contours.

Additionally, we assume that the “angular gradient” of the cardiac wall just inside the outer contour remains small over time and space through,

(15) $$\begin{array}{ll} f_{t}^{\prime \prime }\left(\rho_{n}^{\text{out}}(t),\rho_{n}^{\text{out}}\left(t-1\right)\right)= & \left|d_{n}\left(t,\rho_{n}^{\text{out}}(t)-\epsilon_{\rho }\right)\right)\\ & -\left(d_{n}\left(t,\rho_{n}^{\text{out}}\left(t\;-\;1\right)\;-\;\epsilon_{\rho }\right)\right|, \end{array}$$

and

(16) $$\begin{array}{ll} f_{r}^{\prime \prime }\left(\rho_{n}^{\text{out}}(t),\rho_{n-1}^{\text{out}}(t)\right)= & \left|d_{n}\left(t,\rho_{n}^{\text{out}}(t)-\epsilon_{\rho }\right)\right)\\ & -\left(d_{n}\left(t,\rho_{n-1}^{\text{out}}(t)-\epsilon_{\rho }\right)\right|, \end{array}$$

where *ϵ*_*ρ*_=2 is an experimentally chosen radial offset. Similar feature functions are constructed for the intensity just outside the inner contour.

#### Inner-outer radius feature functions

**Figure 8:**
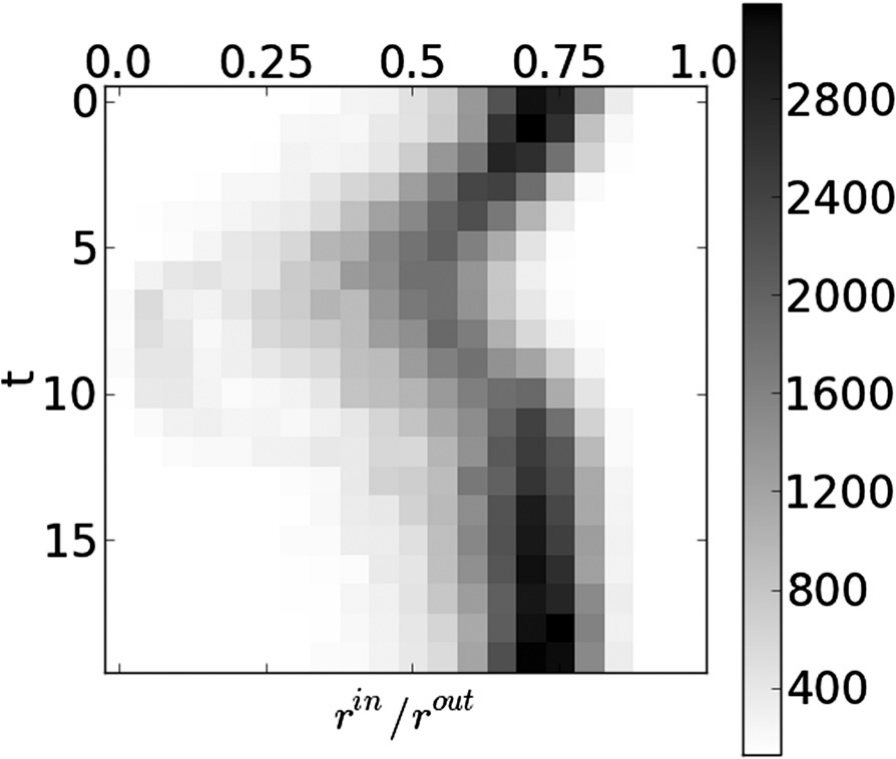
**Inner-outer ratio histogram.** Histogram of relationship between inner and outer radii, $r_{n}^{\text{in}}(t)/r_{n}^{\text{out}}(t)$, against time, generated from a training dataset.

Information on the cardiac structure can further be exploited by using the relationship between the inner and outer contours. The ratio between the inner and outer radii, $r_{n}^{\text{in}}(t)/r_{n}^{\text{out}}(t)$, (and thus the difference in log-space, $\left|\rho_{n}^{\text{out}}(t)-\rho_{n}^{\text{in}}(t)\right|$) is found to contain information on the temporal behaviour, as can be seen in Figure 8. This ratio is related to the wall thickness but is invariant to scaling, which can occur due to differences in patient physiology and MRI magnifications.

**Figure 9:**
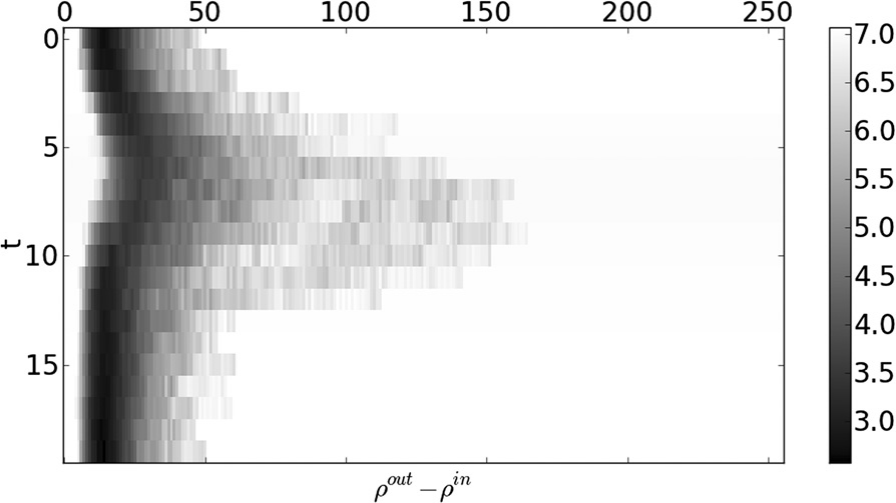
**Inner-outer feature function.** Response of feature, $f_{1}^{\text{cross}}$: difference between inner and outer log radii, $\left|\rho_{n}^{\text{out}}(t)-\rho_{n}^{\text{in}}(t)\right|$ against time.

A probability distribution of the log-radial distance between the inner and outer contours, $P\left(\rho_{n}^{\text{out}}(t)-\rho_{n}^{\text{in}}(t)\right)$, is derived from annotations in a training dataset and used to construct the feature function (see Figure 9),

(17) $$f_{1}^{\text{cross}}\left(\rho_{n}^{\text{in}}(t),\rho_{n}^{\text{out}}(t)\right)=-\log P\left(\overset{\text{out}}{\underset{n}{\rho }}(t)-\rho_{n}^{\text{in}}(t)\right).$$

The relative homogeneity of the cardiac wall can also be exploited by minimizing the variance in intensity of the area between the inner and outer contours through,

(18) $$\;f_{2}^{\text{cross}}\left(\rho_{n}^{\text{in}}(t),\rho_{n}^{\text{out}}(t),d_{n}(t)\right)\;=\;\frac{1}{W}\overset{\rho_{n}^{\text{out}}(t)}{\underset{\rho =\rho_{n}^{\text{in}}(t)}{\sum }}{\left(d_{n}\left(t,\rho \right)\;-\;\mu_{n}\right)}^{2}$$

with the mean wall colour

(19) $$\mu_{n}=\frac{1}{W}\overset{\rho_{n}^{\text{out}}(t)}{\underset{\rho =\rho_{n}^{\text{in}}(t)}{\sum }}d_{n}\left(t,\rho \right),$$

and the wall width

(20) $$W=\rho_{n}^{\text{out}}(t)-\rho_{n}^{\text{in}}(t).$$

### Segmentation

Given a video sequence and trained parameters, we estimate the most probable radii from

(21) $$\rho^{⋆}=\arg\;\underset{\rho }{\max}P\left(\rho |\theta ,D\right),$$

or equivalently the radii with the smallest energy,

(22) $$\rho^{⋆}=\arg\;\underset{\rho }{\min}E\left(\rho |\theta ,D\right).$$

Belief propagation is a popular technique for inferring values for the latent variables in a graphical model. Messages representing cumulative belief are passed between variables and updated according to a specific order or schedule. Backtracking is used to recover a solution. For a tutorial on belief propagation see e.g. [17]. When applied to chains or tree structures, belief propagation is equivalent to dynamic programming, however our model contains many loops and thus requires the iterative loopy belief propagation. For more detail, specifically as applied to this type of edge model, see e.g. [15]. we have chosen a sequential propagation schedule which allows for faster convergence.

#### Propagation schedule

In a parallel propagation schedule all messages are updated simultaneously after each iteration. However, in our experience, the effects from a feature function propagate relatively slowly through the model if a parallel schedule is followed. This is in line with Goldberger and Kfir [18]. For this reason we have chosen a sequential propagation schedule which allows for faster convergence.

For variables representing the inner contour, messages are first propagated in an angular direction (*n*=0,…,*N*−1) and reversed (*n*=*N*−1,…,0) before being propagated to the next temporal frame (*t*=0,…,*T*−1) and back (*t*=*T*−1,…,0). Similar steps are then repeated for the outer contour, taking into account messages passed from the inner contour. This process is repeated for three iterations.

Propagating over the angular direction first places more emphasis on contour continuity than the other radial relationships. The same reasoning is used in the selection of a backtracking order, as discussed below.

#### Message normalization

As features in an undirected graphical model are allowed to take on arbitrary values, propagated messages do not represent marginal probabilities, as is the case in directed graphs. If loopy belief propagation is applied without message normalization, numerical overflow can occur after only a few iterations.

We normalize our messages by subtracting the smallest value in each message before it is propagated, effectively assuring that the minimum value in each message is zero. For a max-product setting this is equivalent to normalizing each message so that the largest value in the message is one.

#### Convergence and optimality

We briefly discuss issues of convergence of message passing and on the choice of an appropriate backtracking order.

Belief propagation is guaranteed to yield a globally optimal result when applied to a tree structured graph [17]. It also converges to a stable fixed point (which is globally optimal) or periodically oscillates when applied to graphs with a single loop [19]. Due to the temporal and inner-outer radius feature functions our graphical model contains many loops (Figure 5).

Convergence for graphs with this many loops is not guaranteed, although if convergence is reached there are theoretical results regarding its optimality. Weiss and Freeman [19] describe the neighbourhood within which the result is optimal.

Through experimentation we find that the inferred segmentation is sensitive to the order of backtracking. We choose to backtrack over all nodes in the inner and outer contours independently. We find that this increases the probability that the inferred inner and outer contours in a single frame form continuous loops.

We have not observed divergence or significant oscillation between configurations in our application.

### CRF parameter estimation

#### Maximum likelihood estimation

Before performing segmentation of a newly observed video sequence, suitable CRF model parameters ***θ*** are needed. One popular approach is to search for parameters that maximize the likelihood of the training annotations, i.e.

(23) $$\theta^{⋆}=\arg\;\underset{\theta }{\max}\;\underset{i}{\prod }P\left(\rho^{\left(i\right)}|D^{\left(i\right)},\theta \right),$$

where *D*^(*i*)^ is a video sequence from a training dataset and ***ρ***^(*i*)^ is its human annotated segmentation.

Often an iterative gradient-based method is used to find adequate parameters. Calculating the probability (or its derivatives) for a specific ***θ***, requires evaluation of the partition function (or its moments). The partition function’s derivative is given by,

(24) $$\begin{array}{ll} \;\frac{\mathrm{\partial Z}\left(\theta ,D_{c}^{\left(i\right)}\right)}{\partial \theta_{c}}\;=\;-\underset{\rho }{\sum } & \left(\;\exp\left(\;-\underset{c^{\prime }}{\sum }\theta_{c^{\prime }}f_{c^{\prime }}\;\left(\rho_{c^{\prime }},\overset{\left(i\right)}{\underset{c^{\prime }}{D}}\right)\;\right)\right)\\ & \left(\cdot \;f_{c}\left(\rho_{c},D_{c}^{\left(i\right)}\right)\;\right). \end{array}$$

Here the sum is over all configurations of ***ρ*** which requires *O*(*M*^2*NT*^) operations. This is one of the significant challenges in applying CRF/MRFs to practical problems as the complexity quickly becomes intractable in general.

Attempts have been made by others to approximate the partition function [20,21], which implies an approximation to the original distribution *P* that we refer to as the distribution $\tilde{P}$.

Apart from these computational difficulties, also note that the approximate nature of loopy belief propagation causes inference to yield a segmentation that is the optimal configuration to a different probability distribution (that we refer to as *Q*) than the one described in Section “The CRF model” (referred to as *P*). The relationship between these distributions is discussed by Weiss and Freeman [19].

The effect of using parameters that maximize the likelihood of the training data under $\tilde{P}$, to infer values from the distribution *Q* is unclear.

However, since we are primarily interested in obtaining parameters that yield good segmentations under inference and less with estimating the “true” model distribution, we investigate an alternative that avoids calculating the partition function.

#### Parameter estimation

Consider a video sequence *D*^(*i*)^ from a training dataset and its human annotated segmentation ***ρ***^(*i*)^. We are interested in obtaining parameters that would lead to a segmentation, ***ρ***^⋆(*i*)^, of the video sequence, that does not significantly differ from the annotated segmentation, ***ρ***^(*i*)^.

Moreover, we wish to find parameters that minimise the errors over the entire training dataset

(25) $$\theta^{⋆}=\arg\;\underset{\theta }{\min}\left(\underset{i}{\sum }e_{i}\left(\rho^{\left(i\right)},\rho^{⋆(i)}\right)\right)$$

where the inferred segmentation of a video sequence from loopy belief propagation is

(26) $$\rho^{⋆(i)}=\arg\;\underset{\rho }{\max}\;Q\left(\rho |\theta ,D^{\left(i\right)}\right).$$

The function *e*_*i*_ is a measure of the differences between two segmentations (i.e. the ground-truth and inferred contours). The landmark error, i.e. the average of the shortest distance between each point on the ground truth contour and the inferred contour, is used by Andreopoulos *et al*[9] in their evaluation. Due to its non-symmetry, i.e. *e*_*i*_(***ρ***^*A*^,***ρ***^*B*^)≠*e*_*i*_(***ρ***^*B*^,***ρ***^*A*^), we have observed that inferred contours that minimize this error tend to be very jagged. For this reason we use the average of the “ground truth to inferred” error and the “inferred to ground truth” error during training, i.e. $\frac{e_{i}\left(\rho^{A},\rho^{B}\right)+e_{i}\left(\rho^{B},\rho^{A}\right)}{2}$.

Powell’s method [22] is a popular technique for searching for the local minimum of a function and is used here to find suitable parameters. Powell’s method avoids calculating a gradient through a bidirectional line search along a vector in a list of vectors. The list is updated by the displacement after each improving iteration. In our case convergence requires about 300 iterations.

To compensate for the approximate nature of inference and avoid calculating the partition function, a gradient-free approach is thus followed where the segmentation error is treated as a “black box”. This allows us to integrate the approximate inference process into the training stage.

### Implementation

The majority of the software is implemented in the Python programming language with belief propagation implemented in C. Cordero-Grande et al. [13] reported segmentation of a single 4D video sequence in approximately 56 minutes on a single 800MHz CPU with 4MB cache from their MRF. For a similar number of images (12 slice positions with 20 frames each) on a single 3400MHz CPU with 8MB cache we can report a radial segmentation time of ~2 minutes. We have not included the time to estimate the centre point positions, which is approximately an additional minute for each 20 frame video sequence.

Our radial inference technique is thus approximately six times faster (taking into account the faster CPU), which can be attributed to their use of a Gibbs sampler. Note also that the number of radial points we use to represent a contour and its radial discretisation are 128 and 256, respectively. This is significantly larger than the values of 7 and 31 used by Cordero-Grande et al., especially when comparing the size of the resulting configuration space.

## Results

In this section we analyse the segmentations produced by our process and compare them to those of a few existing techniques. The reader is urged to view the video sequences in Additional file 1 or on our website at http://dip.sun.ac.za/~janto. Additional file 2 contains the automatic segmentations of all end-systole and end-diastole frames for the Sunnybrook evaluation dataset.

We evaluate our model on two datasets. Our segmentation process is trained and analysed on the York dataset [9] with respect to segmentation behaviour and its sensitivity to placement of the initial centre point. This dataset contains ground truth annotations for all frames and therefore contains important temporal information. For comparison with other authors our technique is evaluated on the Sunnybrook [5] MRI dataset. This dataset is not annotated for all frames, but has the advantage that more authors have used this set to report their results.

In addition to the landmark distance, we use two other similarity measures that have gained widespread use: the Dice metric and Average Perpendicular Distance (APD). The Dice similarity represents the percentage of overlap between two segmented surfaces and the Dice error is the percentage of non-overlap, i.e. one minus the Dice similarity. The APD is calculated as the average of the perpendicular distance from each point on the reference contour to the target contour. The APD is therefore similar to the non-symmetric landmark distance.

### York cardiac segmentation dataset

In this section we evaluate our model on the MRI York dataset [9] provided by the Department of Diagnostic Imaging of the Hospital for Sick Children in Toronto and annotated by Andreopoulos of York University. The dataset contains video sequences from 33 subjects, all under the age of 18, displaying a variety of heart abnormalities such as cardiomyopathy, aortic regurgitation, enlarged ventricles and ischemia. We split the dataset into three cross-validation sets: 11 subjects for training of the edge classifier, 11 subjects for CRF parameter estimation and 11 subjects for evaluation. Each set effectively contains approximately 100 video sequences and each video contains 20 frames at different z-axis slice positions. The inner and outer contours are manually annotated for all frames and are used as the ground truth in our experiments.

For efficiency we only use video sequences of the sixth z-axis slice of each patient to estimate the CRF parameters. These slices do not necessarily coincide with the mid ventricle for all patients. The number of slices where the ventricle is visible differs between patients and is likely dependent on patient pathology. A different choice of training data is thus likely to have a significant effect on the results of patients with, for example, hypertrophy.

#### Segmentation analysis

**Figure 10:**
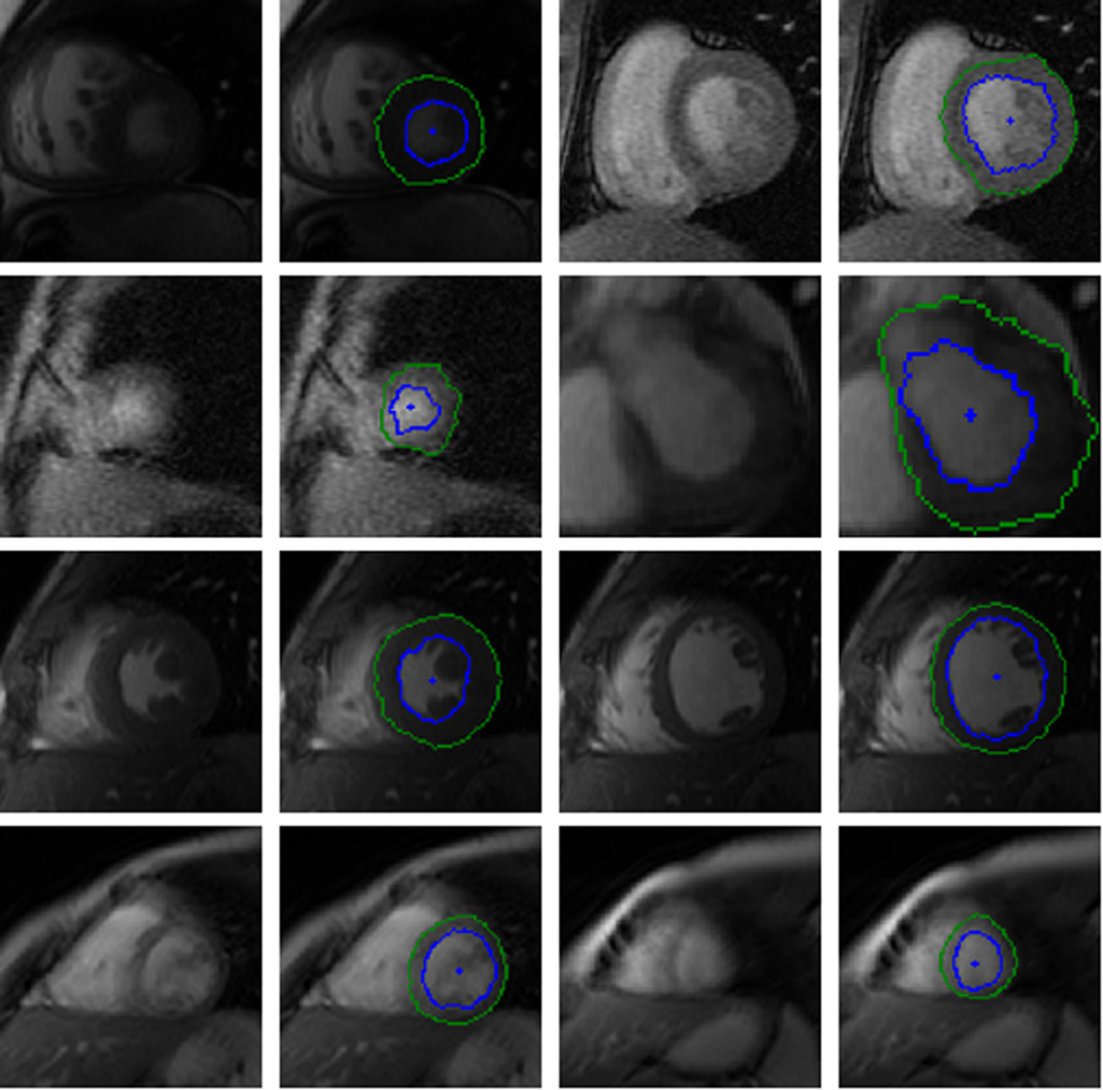
**York automatic segmentations.** Selection of images and their automatically segmented contours (inner contour is blue and outer is green) inferred from a testing set. The blue dot in the middle of the blood pool is the estimated centre point.

Figure 10 illustrates our results on a selection of images from the testing dataset. A visual inspection indicates that, for the majority of video sequences, our automated annotations are in line with expected behaviour with regard to shape, position and motion. Of particular interest is the inclusion of the papillary muscles inside the inner contour. In many of these cases the segmentation process is able to use local shape and temporal behaviour to identify the inner contour even though the edge is weak or absent.

We also observe robustness to some noisy images. These examples suggest that in some of these images where the automatic segmentation differs from the ground truth, the automatic segmentation is superior to the manual approach. This is attributed to the fact that the automated system is able to integrate temporal behaviour, something that is an arduous task for a human.

**Figure 11:**
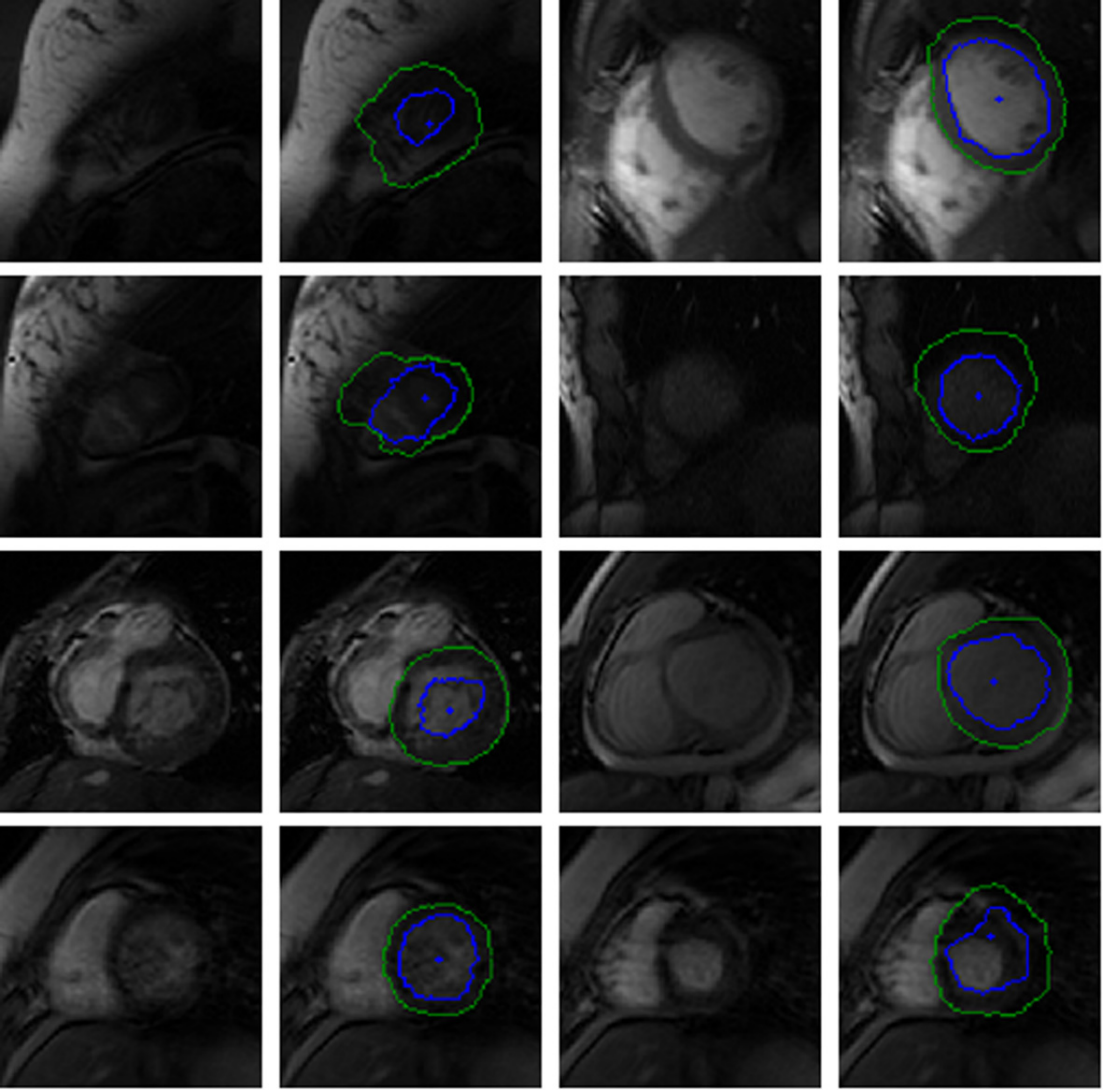
**Bad quality York automatic segmentations.** Selection of incorrectly segmented contours (inner contour is blue and outer is green) inferred from a testing set. The blue dot in the middle of the blood pool is the estimated centre point.

Figure 11 contains a selection of images that are incorrectly segmented. These are primarily due to a disappearing blood pool, centre point initialization outside the endocardium, or low contrast between the endocardium and cardiac wall.

**Figure 12:**
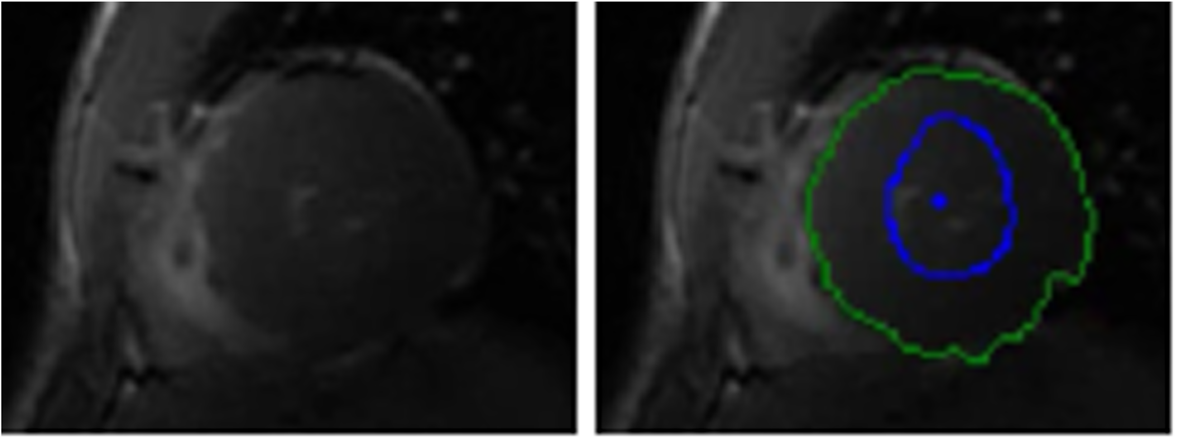
**Disappearing blood pool.** Incorrect automatically segmented contours (inner contour is blue and outer is green) of Subject 8 due to a disappearing blood pool. The blue dot in the middle is the estimated centre point.

On further inspection of the York dataset, the blood pool disappears from view in some video sequences of Subject 8 (refer to Figure 12) which is diagnosed with ventricular hypertrophy (enlarged cardiac wall thickness). It is also noted by the annotator of the dataset [9] that this yields bad segmentations in their work. Images of Subject 27 also has relatively low contrast between the endocardium and the cardiac wall. We therefore regard Subject 8 and 27 as outliers and remove them from the dataset. This significantly improves inner contour accuracy as indicated in Table 1.

**Table 1:** Comparison of contour errors

**Authors**	**Technique**	**Inner contour**	**Outer contour**
		**error [mm]**	**error [mm]**
Our method	CRF	1.57	1.78
Our method (without subjects 8 and 27)	CRF	1.49	1.74
Andreopoulos and Tsotsos [9]	AAM	1.43	1.51
Üzümcü [23]	Landmark tracking	1.86	1.77
Jolly [14]	Shortest path	2.44	2.05
Cordero-Grande et al. [13]	Edge MRF	1.37	1.22
Lorenzo-Valdés et al. [24]	Surface MRF	2.99	2.21

Average segmentations errors as reported by different authors. These results are not strictly comparable since they are based on different datasets and the error criteria differ. Refer to the individual papers for more detail.

**Figure 13:**
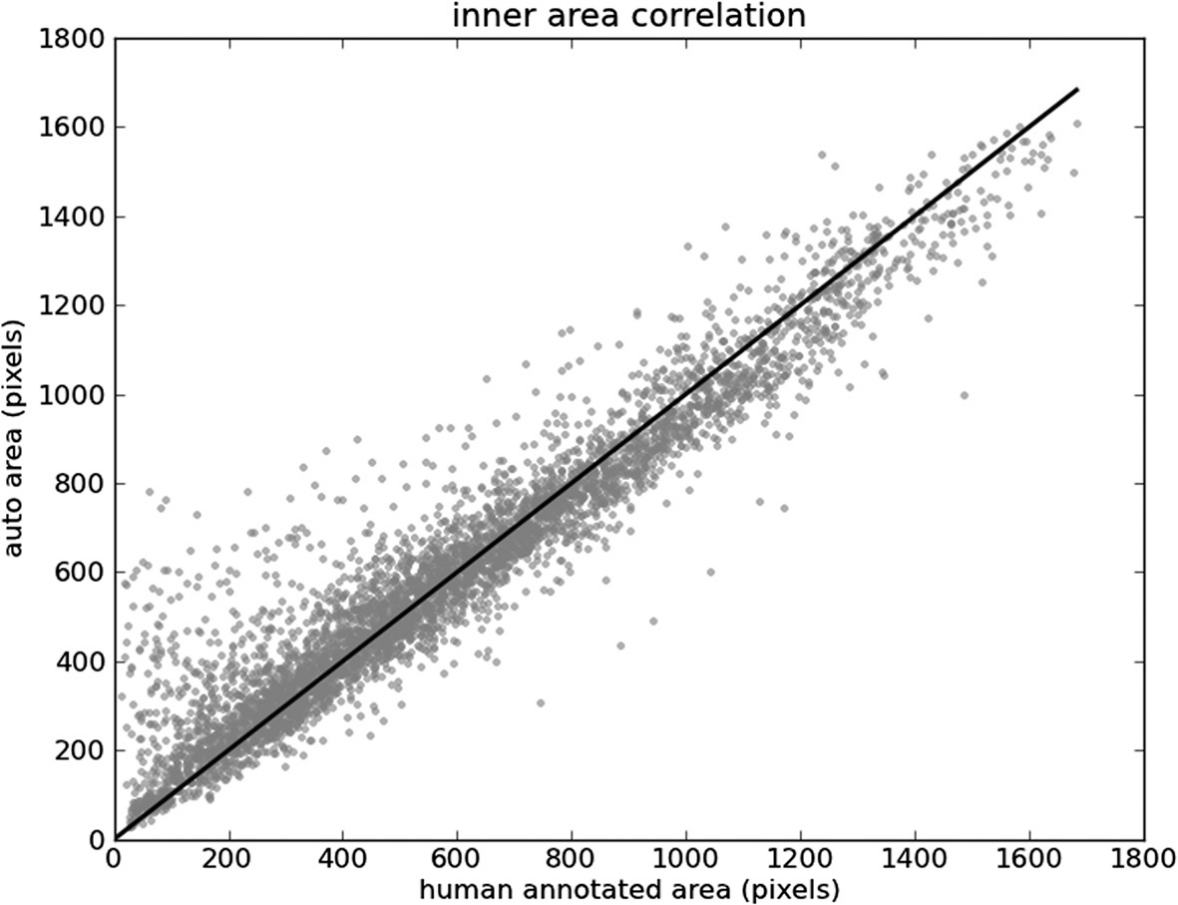
**Inner contour areas.** Areas inside inner contours of human annotation against the areas inside automated segmentation of testing data.

**Figure 14:**
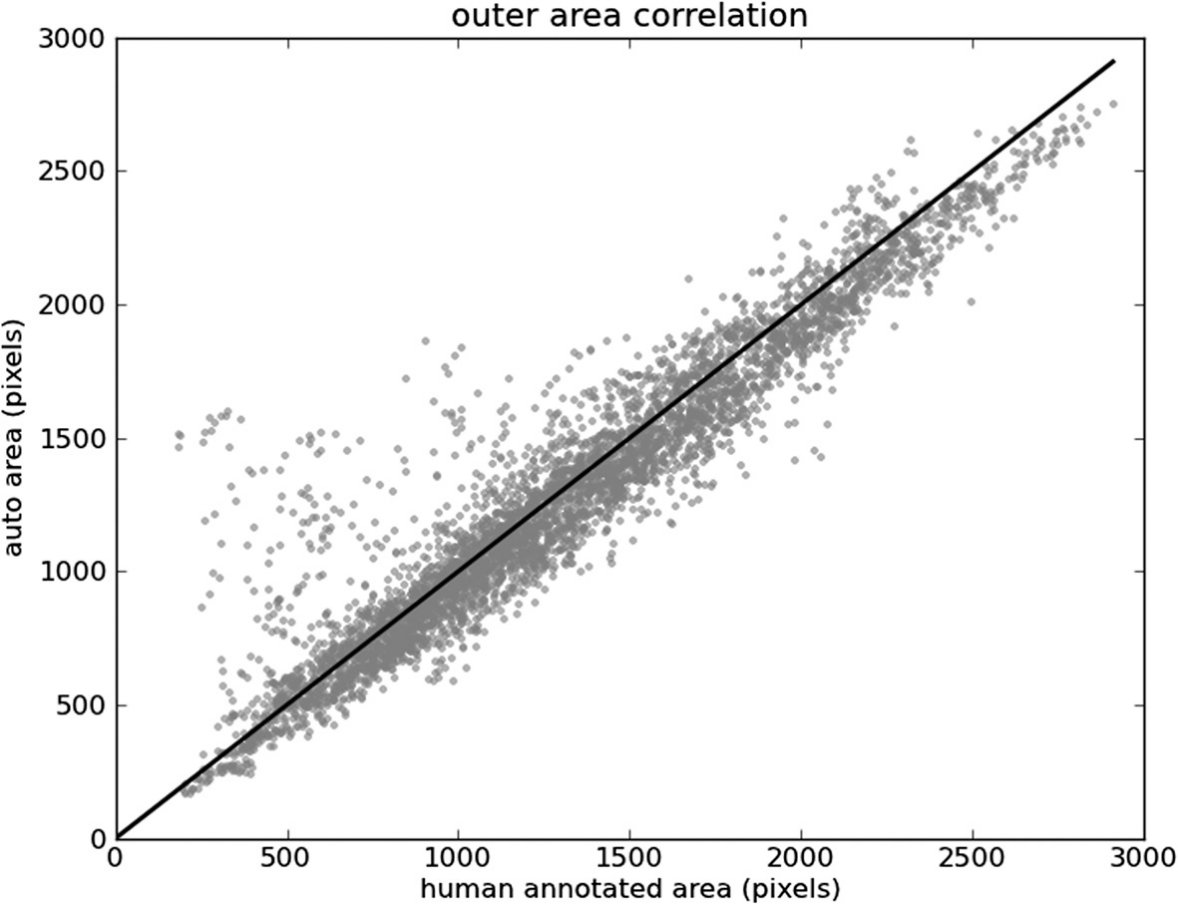
**Outer contour areas.** Areas inside outer contours of human annotation against the areas inside automated segmentation of testing data.

The areas inside the contours of the automatic annotations are plotted against the areas inside the human annotated ground truth in Figures 13 and 14. For small inner contours our technique often yields segmentations larger than the ground truth. This can be attributed to the automated segmentations being more “inclusive” of the papillary muscles, which can significantly effect small contours. Our technique also provides slightly smaller outer contours. A comparison with the ground truth indicates that our segmentation is often temporally smoother. This is attributed to the human annotator segmenting one frame at a time, and thereby largely disregarding temporal behaviour.

**Figure 15:**
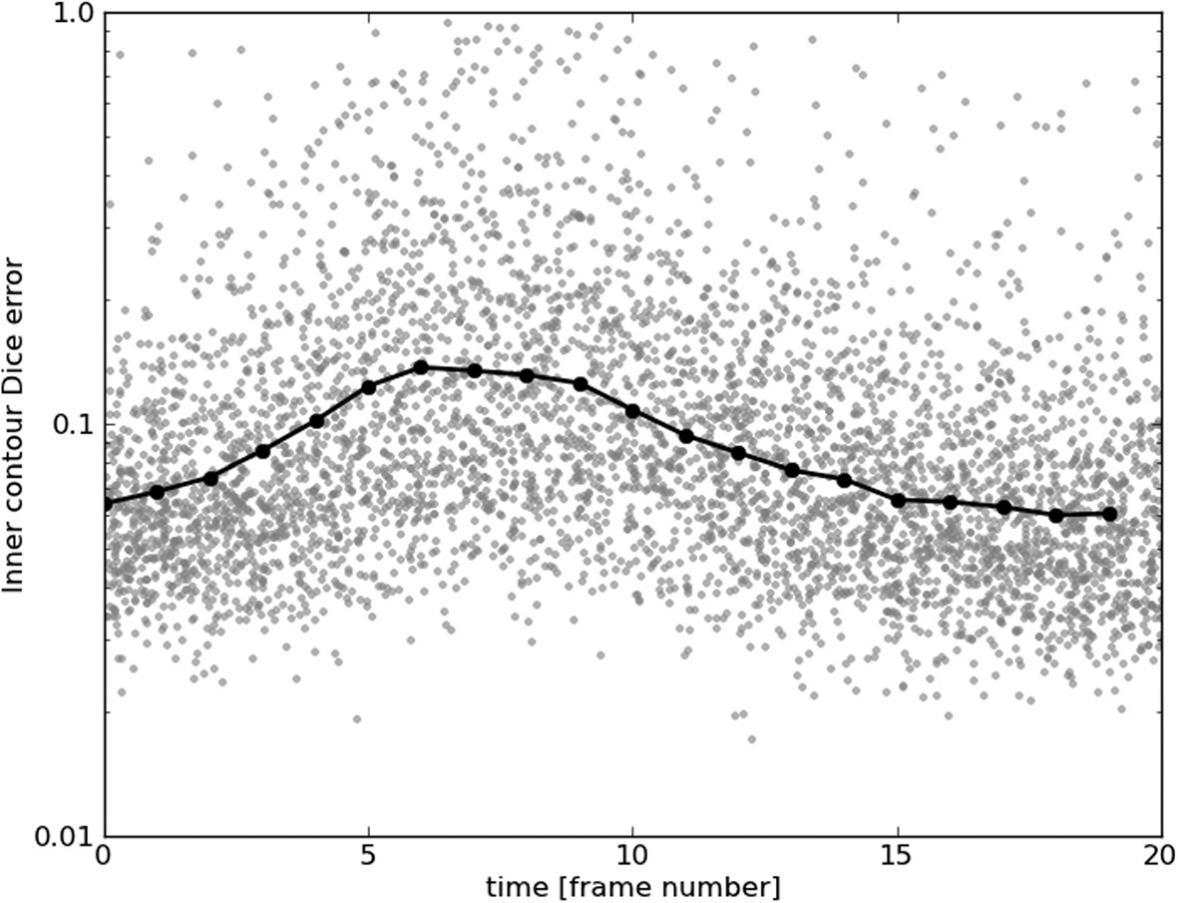
**Inner Dice errors over time.** Inner contour Dice errors over time. For illustrative purposes, a random real value between zero and one was added to each frame number. The geometric mean for each frame number is indicated by a black line.

**Figure 16:**
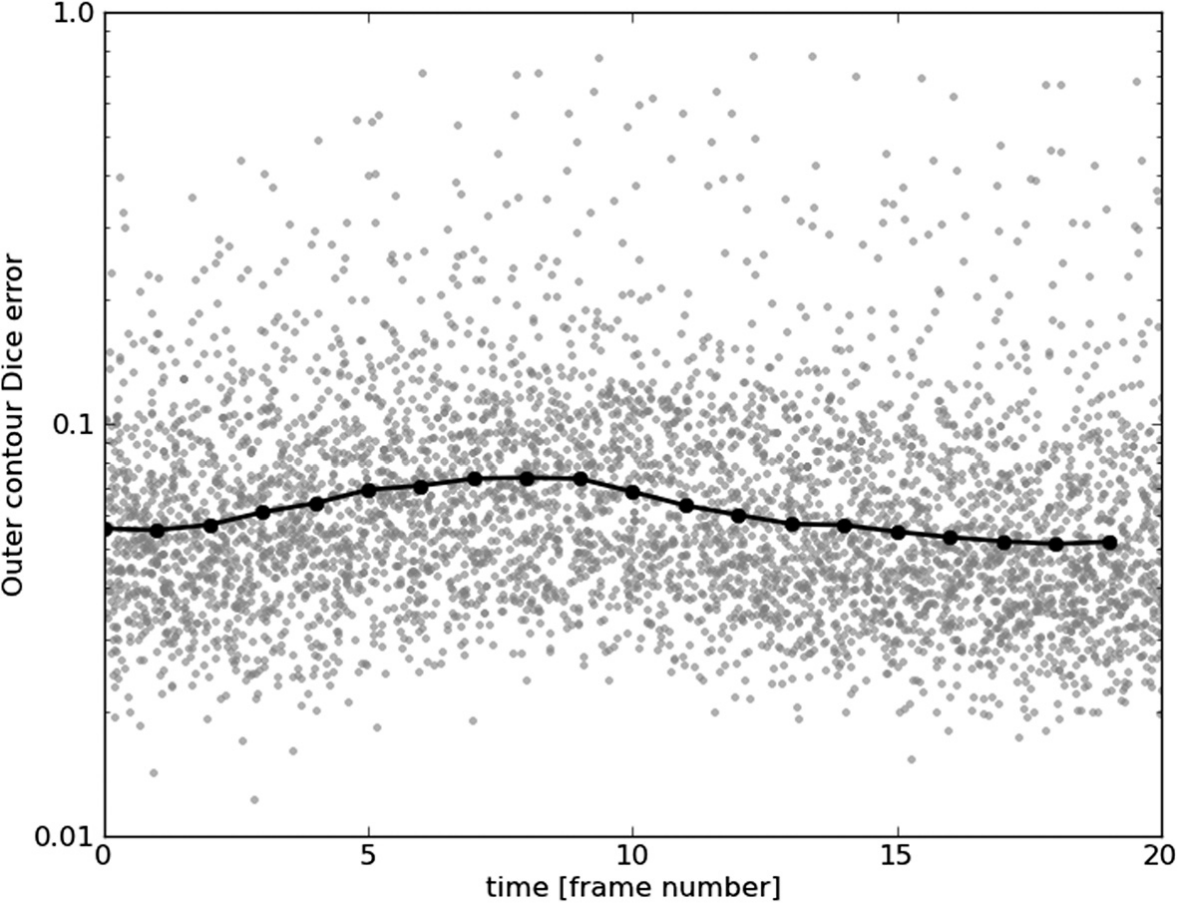
**Outer Dice errors over time.** Outer contour Dice errors over time. For illustrative purposes, a random real value between zero and one was added to each frame number. The geometric mean for each frame number is indicated by a black line.

**Figure 17:**
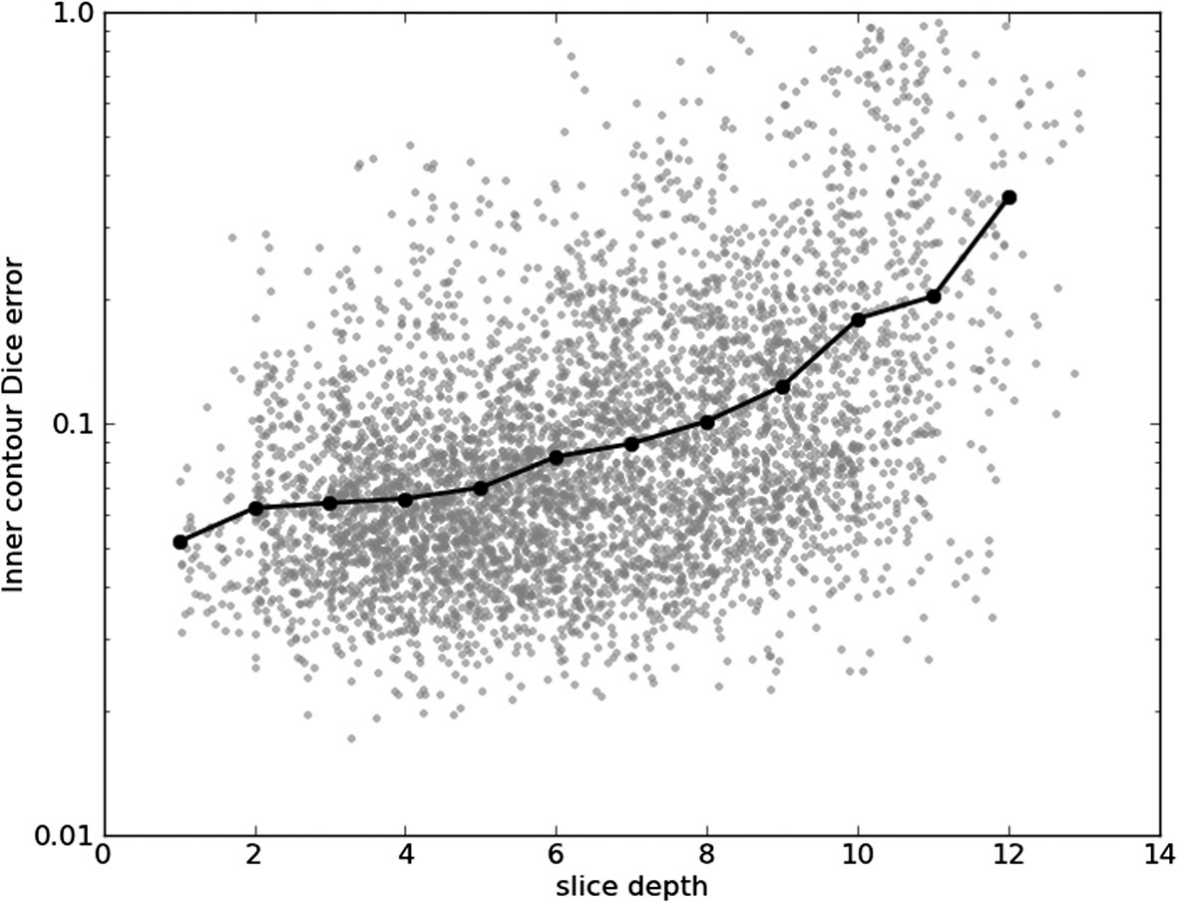
**Inner Dice errors over slices.** Inner contour Dice errors over different slices. For illustrative purposes, a random real value between zero and one was added to each slice depth. The geometric means for the slice positions are indicated by the black line.

**Figure 18:**
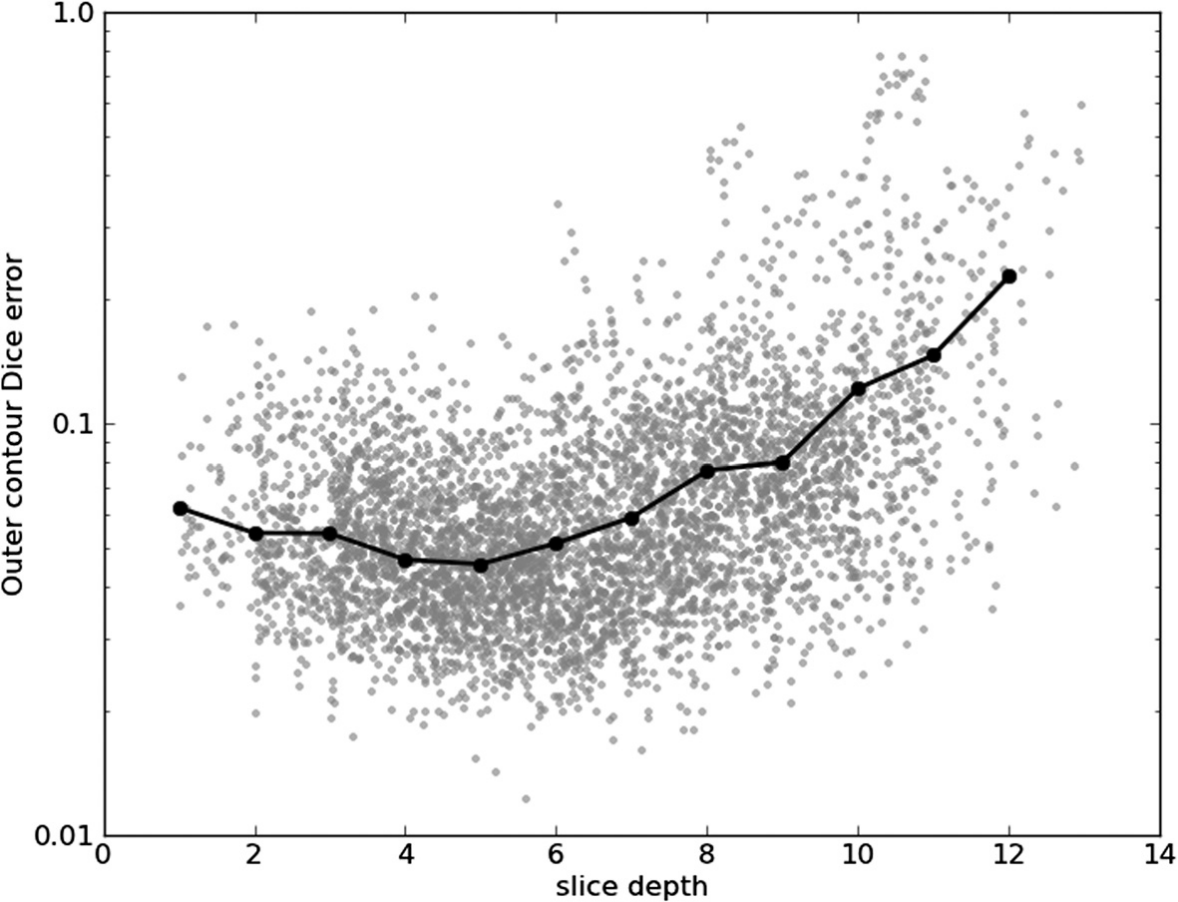
**Outer Dice errors over slices.** Outer contour Dice errors over different slices. For illustrative purposes, a random real value between zero and one was added to each slice depth. The geometric means for the slice positions are indicated by the black line.

Figures 15, 16, 17 and 18 show frame Dice errors against time and slice position for the inner and outer contours. The vertical axes of these graphs are logarithmically scaled. The geometric means (arithmetic mean in the log-scale) in these figures are indicated by solid black lines. We observe that the majority of incorrect segmentations occur during end-systole (*t*≈8) and spatially lower slices (depth>9) where the blood pool is at its smallest (sometimes completely disappearing from view). In these frames papillary muscles are most visible, obscuring not only the border, but a significant part of the endocardium.

Table 1 contains segmentation errors of the inner and outer contours as reported by a selection of different authors. We report our results as the landmark error [9], i.e. the average of the shortest distances between the points on the ground truth contour and the inferred contour. These results are, however, from different datasets and there are also subtle, but important, differences in the error criteria. Refer to the individual papers for more detail. This table should therefore only be used as a rough comparison to other techniques. For a more comprehensive summary of reported errors see [25]. The Sunnybrook dataset is used in a later section to more fairly compare our technique to others.

#### Sensitivity to initial centre point placement

**Figure 19:**
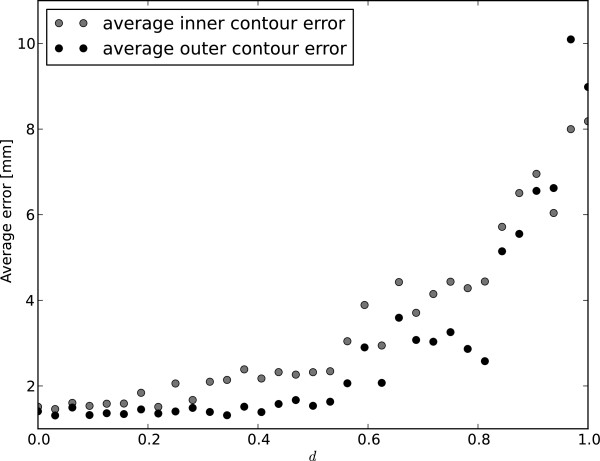
**Centre point robustness.** Sensitivity of segmentation with regard to incorrect centre point in first frame. The distance *d* is the fractional position between the ground truth centre point and the ground truth inner contour. See Section “Sensitivity to initial centre point placement” for more detail.

Figure 19 illustrates the sensitivity of the segmentation to incorrect placement of the initial centre point, *c*(0). For each video sequence, an initial centre point is generated at a fractional distance, *d*, between the ground truth centre and a randomly selected point on the ground truth inner contour. The CRF parameters are not retrained on these imperfectly placed centre points, which would possibly allow the system to weigh shape information less and thus improve results.

As can be seen from Figure 19, the segmentations of the inner and outer contours remain relatively stable while *c*(0) is within the inner 20% of the endocardium. When placed at approximately 50% between the ground truth centre point and inner contour, the spatial continuity assumption of (12) is violated enough that the quality of the inferred contours begin to deteriorate significantly.

### Sunnybrook cardiac segmentation dataset

The Sunnybrook Cardiac MR Database [5] is provided by the Sunnybrook Health Sciences Centre and was used for the 2009 MICCAI Cardiac MR Left Ventricle Segmentation Challenge. The dataset contains 45 subjects, with an average age of 61, with diverse morphologies (Heart failure with and without infarction, LV hypertrophy, and healthy subjects) and is manually segmented by a cardiologist. The inner contours are annotated only at end-diastole and end-systole, while the outer contours are annotated only at end-systole. A ground truth segmentation of the intermittent frames is therefore not available, making extraction of temporal information for this dataset difficult.

Due to the sparsity of annotations in this dataset, all feature functions are re-used as derived from the York dataset. The model used to segment the Sunnybrook data therefore includes features derived from the trained edge classifiers, temporal behaviour and inner-outer relationships of the York dataset. Only the CRF parameters (i.e. the relative importance of the features) are retrained on a training subset of the Sunnybrook data. To further compensate for the relatively few examples, and thus avoid over-fitting, a very weakly weighted L1-norm parameter regularization term ($\underset{d}{\sum }\left|\log\theta_{d}\right|$) is added to the objective function. Since this term is applied to the log of the parameters, it effectively penalizes the specialization on features by the optimizer. A detailed analysis of the effects of parameter regularization in our application is beyond the scope of this article.

**Figure 20:**
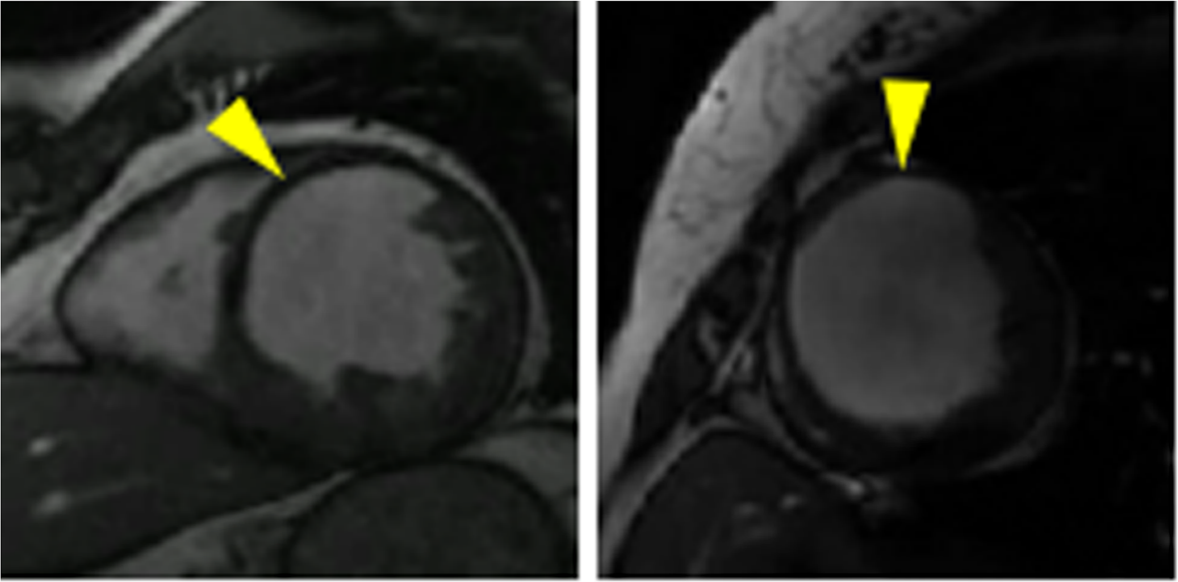
**Sunnybrook wall thickness.** Examples from the Sunnybrook dataset with thin cardiac walls, as indicated by the yellow arrows.

A notable difference from the York dataset is the presence of images of patients with heart failure, where the cardiac wall is exceptionally thin, as indicated by the yellow arrows in Figure 20. To compensate we modified the belief propagation beam search by reducing the minimum allowed difference between the inner and outer variables (i.e. wall thickness) from 10 to 2. We also reduced the radial offset used in the features described by (15) and (16) from *ϵ*_*ρ*_=2 pixels to *ϵ*_*ρ*_=1, to adequately capture the wall colour when very thin.

This modification suggests that there are important model parameters that are dependant on the patient pathology. A practical segmentation tool could allow the operator the option to provide a prior diagnosis or more fine grained control over some settings.

During training of the edge classifier from the York dataset, it was assumed that the extracted radial window, ***v***_*ρ*_, contains an edge if the annotated edge is within two radial distances from the middle of the window. This assumption is problematic if used to train the edge classifier on the Sunnybrook dataset. A small wall thickness, as is common in this dataset, would cause both the inner and outer contours to fall within this criteria, resulting in inconsistencies in the training examples and thus weakening the resulting edge detector. To train a classifier on this dataset it would thus be necessary to be more strict with regard to the minimum radial distance.

#### Segmentation analysis

**Figure 21:**
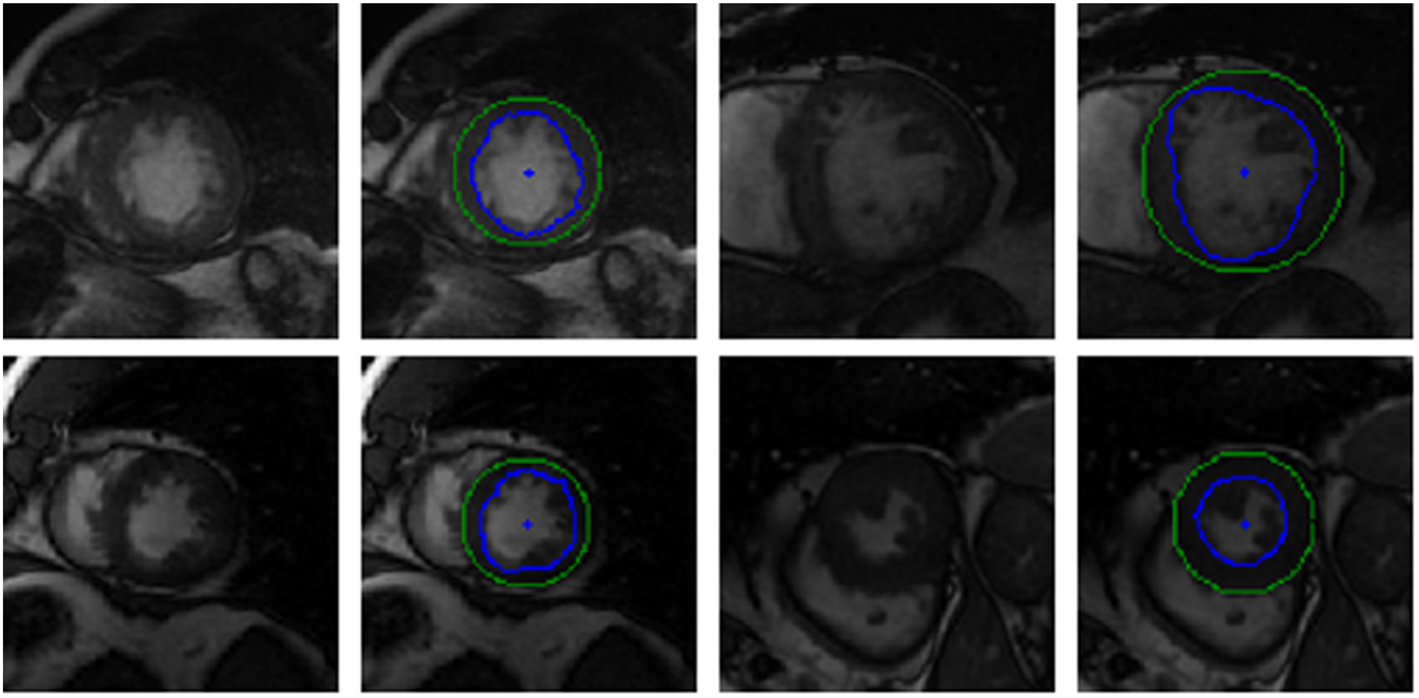
**Sunnybrook automatic segmentations.** Selection of images and their automatically segmented contours (inner contour is blue and outer is green). The blue dot in the middle of the endocardium is the estimated centre point.

Figure 21 contains a selection of images from this dataset and their automatic segmentations. See also Additional file 2. A qualitative examination of the results suggests that segmentations are generally of good quality; the papillary muscles are included even if they obscure the endocardium border.

**Figure 22:**
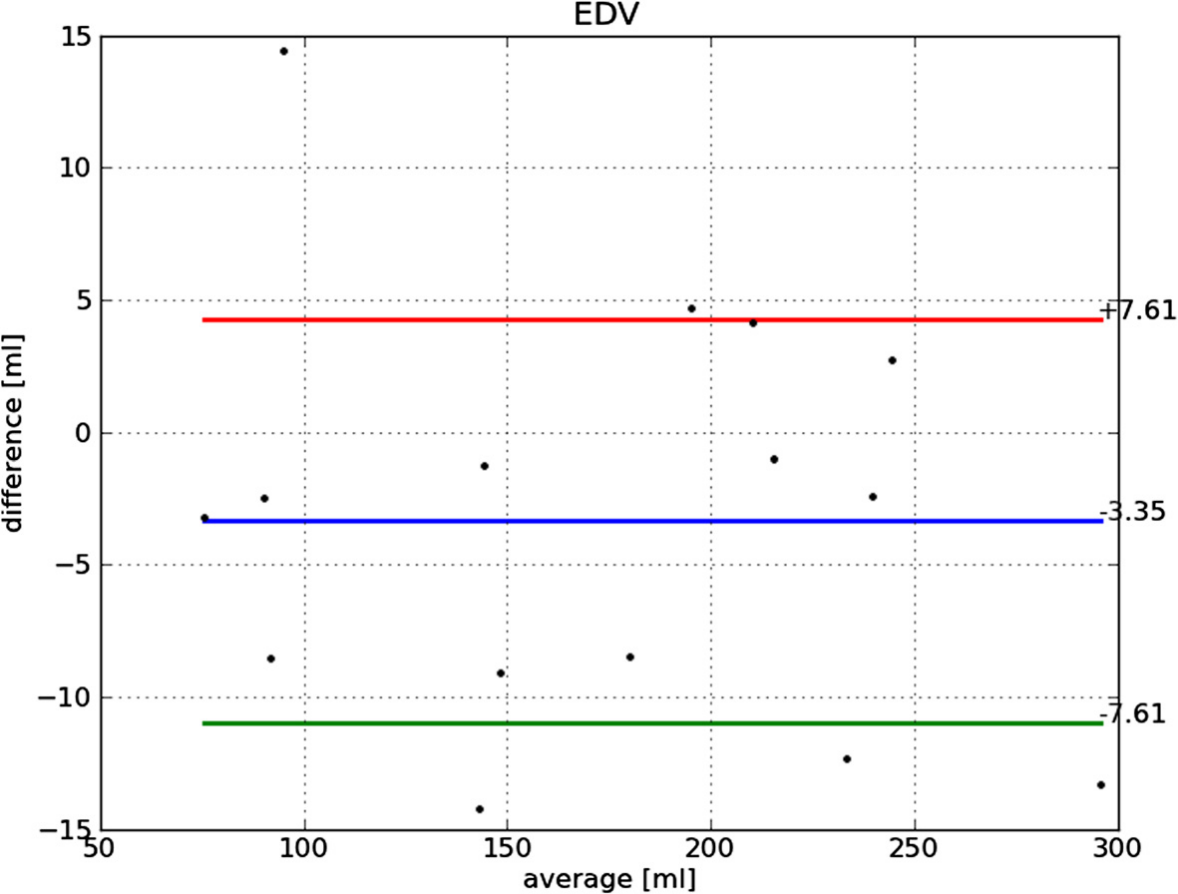
**Bland-Altman plot of EDV.** Bland-Altman plot of *automatically determined* minus *ground truth* end-diastole volume including mean difference and standard deviation lines.

**Figure 23:**
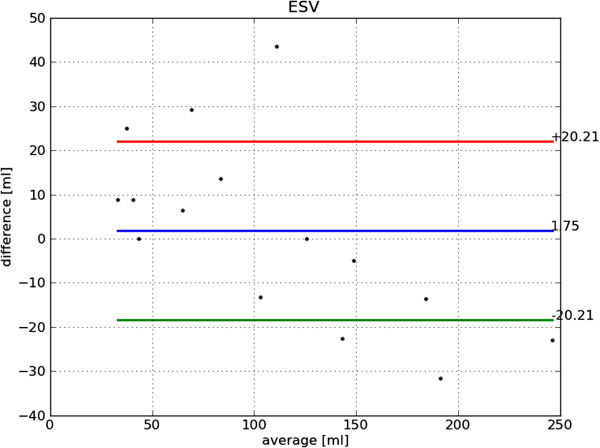
**Bland-Altman plot of ESV.** Bland-Altman plot of *automatically determined* minus *ground truth* end-systole volume including mean difference and standard deviation lines.

**Figure 24:**
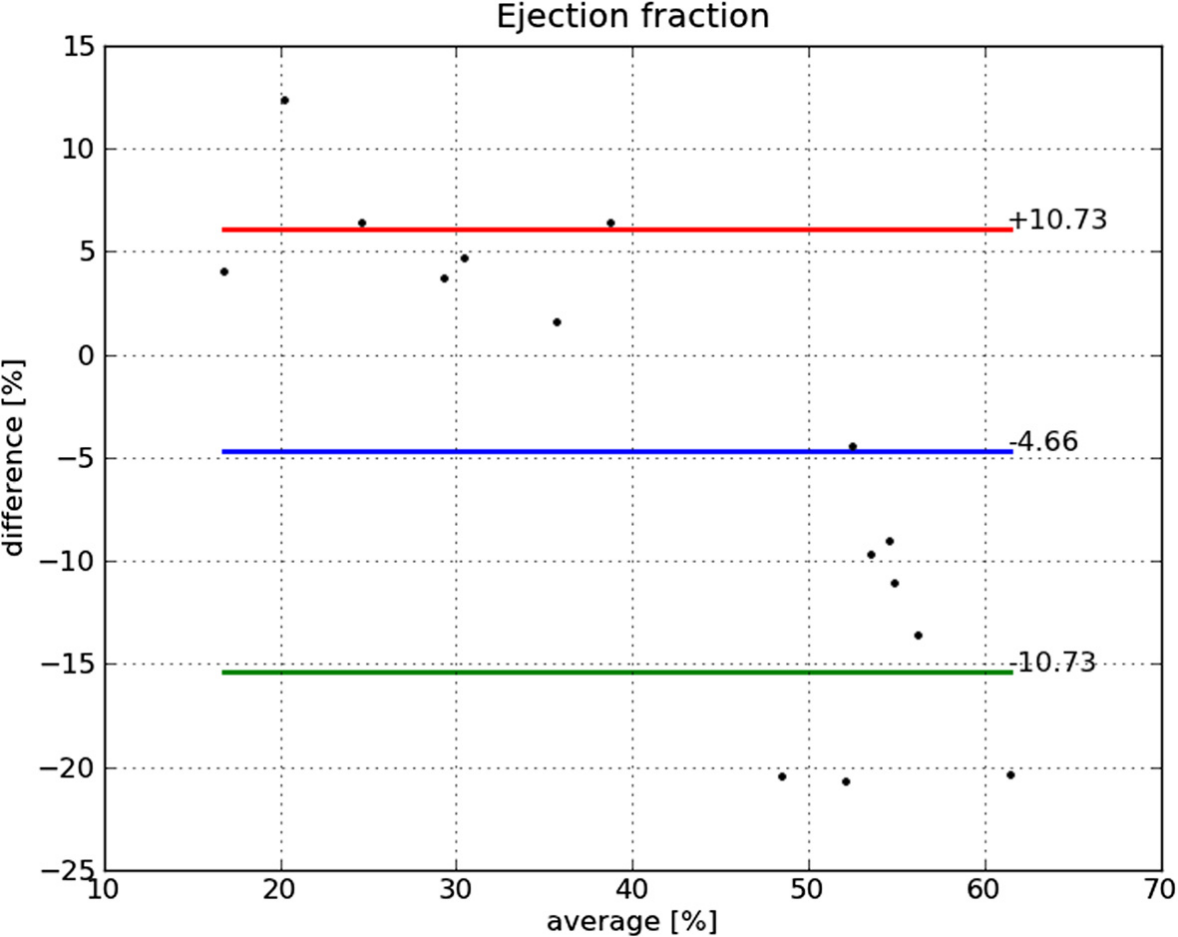
**Bland-Altman plot of EF.** Bland-Altman plot of *automatically determined* minus *ground truth* ejection fraction including mean difference and standard deviation lines.

**Figure 25:**
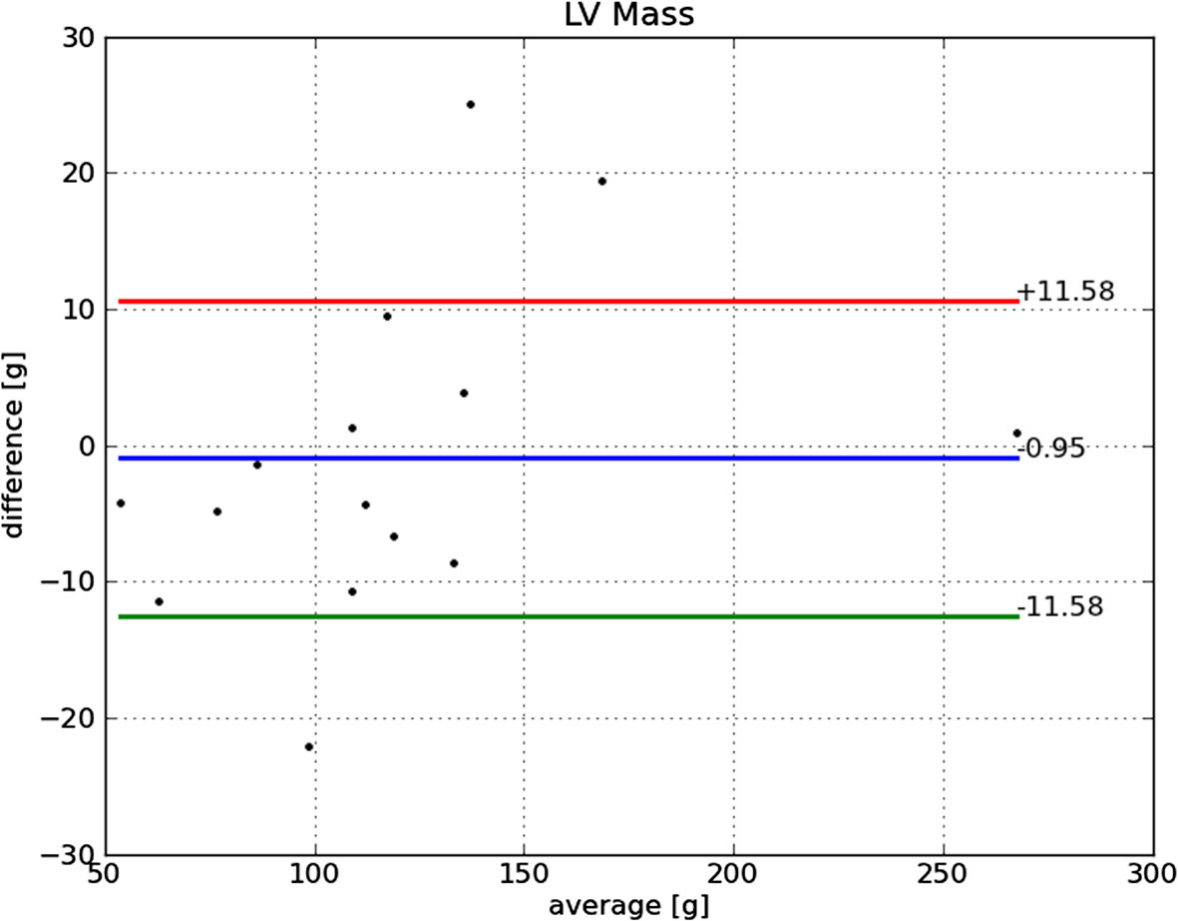
**Bland-Altman plot of LV mass.** Bland-Altman plot of *automatically determined* minus *ground truth* LV mass including mean difference and standard deviation lines.

Bland-Altman plots of end-diastole volume, end-systole volume, ejection fraction and mass are shown in Figures 22, 23, 24 and 25, respectively. The end-diastole volumes, as predicted by our technique and as annotated by the human specialist, agree with a small bias and variance (−3.35±7.61 ml). End-systole volumes agree with a small bias, but a relatively large variance (1.75±20.21 ml). This leads to a relatively small bias, but relatively large variance in the agreement of the calculated ejection fractions (−4.66±10.73%). The calculated left ventricle mass has a small bias and variance (−0.95±11.58 g).

**Figure 26:**
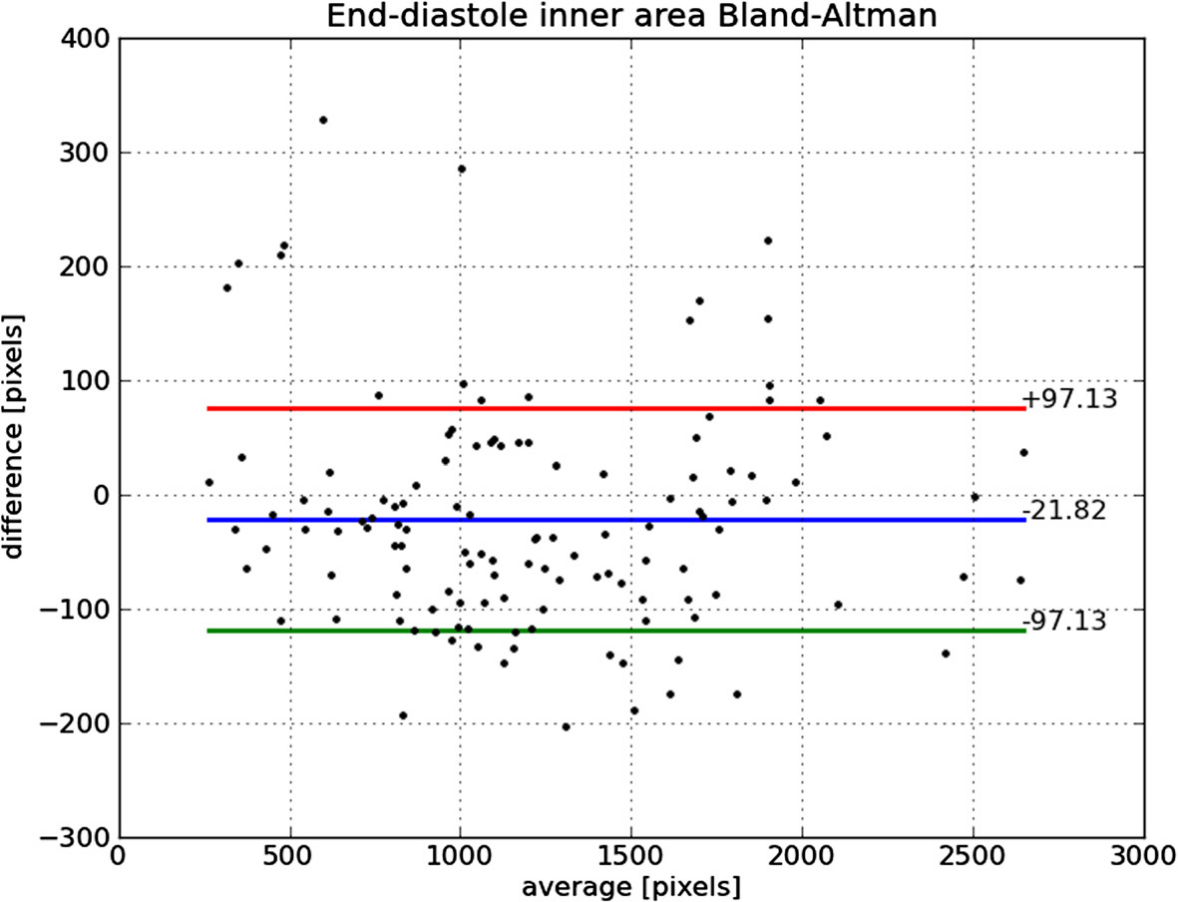
**Bland-Altman plot of ED area.** Bland-Altman plot of *automatically determined* minus *ground truth* end-diastole area including mean difference and standard deviation lines.

**Figure 27:**
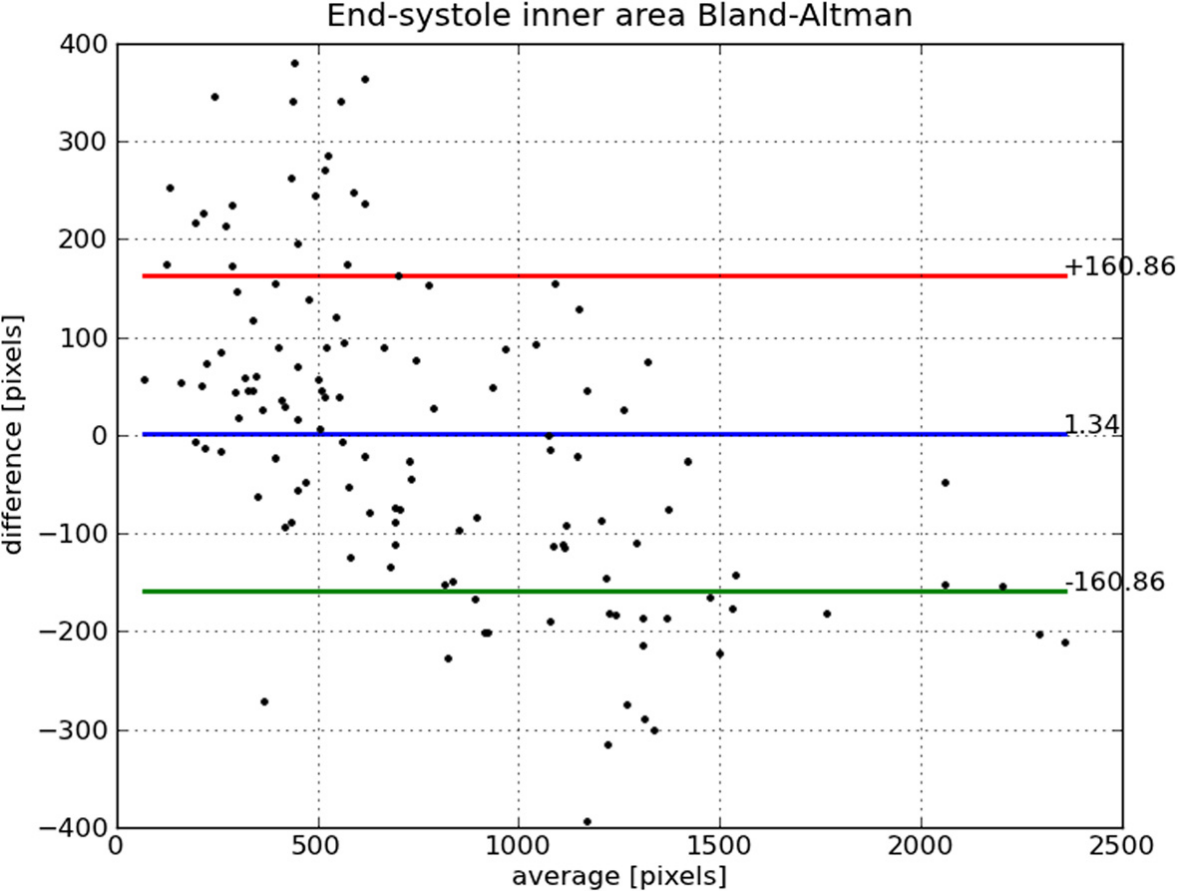
**Bland-Altman plot of ES area.** Bland-Altman plot of *automatically determined* minus *ground truth* end-systole area including mean difference and standard deviation lines.

The end-diastole volume in Figure 22 and Bland-Altman plot of end-diastole inner contour area in Figure 26 also illustrate the algorithm’s tendency to yield contours smaller than the ground truth at end-diastole. The Bland-Altman plot of end-systolic volume in Figure 23 and end-systolic inner contour area in Figure 27 illustrate that small volumes are overestimated and large volumes underestimated.

Table 2 contains a summary of Dice errors and APD as reported by various authors on the Sunnybrook dataset during the challenge [5] including our results, before and after parameter retraining. Prior to retraining, results are comparable but slightly worse than the top performing challenge entries. After parameter re-estimation on the training subset our results outperform the entries on the evaluation set in terms of the inner contours’ Dice metric. Our average Dice similarity of the outer contours is comparable to the best performing entries in the challenge. Our average APD for the inner and outer contours are equal to or smaller than reported by any of the authors in the challenge.

**Table 2:** Comparison of Sunnybrook Dice errors

**Authors**	**Dice similarity**		**APD (mm)**	
	**Inner**	**Outer**	**Inner**	**Outer**
Our method (trained on York)	0.87	0.92	2.70	2.23
**Our method (after retraining)**	**0.91**	**0.93**	**1.84**	**1.95**
Marak et al. [26]	0.86	0.93	2.6	3.0
Lu et al. [27]	0.89	0.94	2.07	1.91
Wijnhout et al. [28]	0.89	0.93	2.29	2.28
Casta et al. [29]	-	0.93	-	2.72
O’Brien et al. [30]	0.81	0.91	3.73	3.16
Constantinides et al. [31]	0.89	0.92	2.35	2.04
Huang S. et al. [32]	0.89	0.94	2.10	1.95
Jolly [14]	0.88	0.93	2.44	2.05

Average Dice similarity metric and Average Perpendicular Distance (APD) for our segmentation of the Sunnybrook validation set (before and after training) and those reported by the different challenge entries.

Table 3 provides a more detailed report on the resulting segmentations of the patients in the evaluation set, as generated by the evaluation code provided with the dataset. The table also indicates the percentage of good contours for each subject, i.e. those with an APD smaller than 5 mm.

**Table 3:** Sunnybrook results

**Patient**	**Good (%)**		**APD (mm)**		**Dice similarity**	
	**Inner**	**Outer**	**Inner**	**Outer**	**Inner**	**Outer**
SC-HF-I-05	100	100	1.52	1.86	0.94	0.95
SC-HF-I-06	100	100	1.66	1.45	0.92	0.95
SC-HF-I-07	100	100	2.37	2.79	0.89	0.90
SC-HF-I-08	95	100	1.68	1.37	0.93	0.96
SC-HF-NI-07	100	100	2.21	2.20	0.91	0.93
SC-HF-NI-11	100	100	1.70	1.25	0.93	0.96
SC-HF-NI-31	100	100	2.06	1.58	0.91	0.95
SC-HF-NI-33	94	100	1.68	1.64	0.91	0.94
SC-HYP-06	92	100	1.67	2.05	0.90	0.92
SC-HYP-07	69	100	1.39	1.83	0.93	0.94
SC-HYP-08	68	100	2.27	2.33	0.90	0.93
SC-HYP-37	69	71	2.21	2.44	0.86	0.91
SC-N-05	93	100	1.67	2.45	0.89	0.89
SC-N-06	100	86	1.76	2.12	0.89	0.91
SC-N-07	100	100	1.79	1.95	0.88	0.90
mean	92	97	1.84	1.95	0.91	0.93
std deviation	12.4	8.0	0.30	0.44	0.02	0.02

Patient specific Average Perpendicular Distance (APD), and Dice similarity between annotations and ground truth of the Sunnybrook validation set.

**Figure 28:**
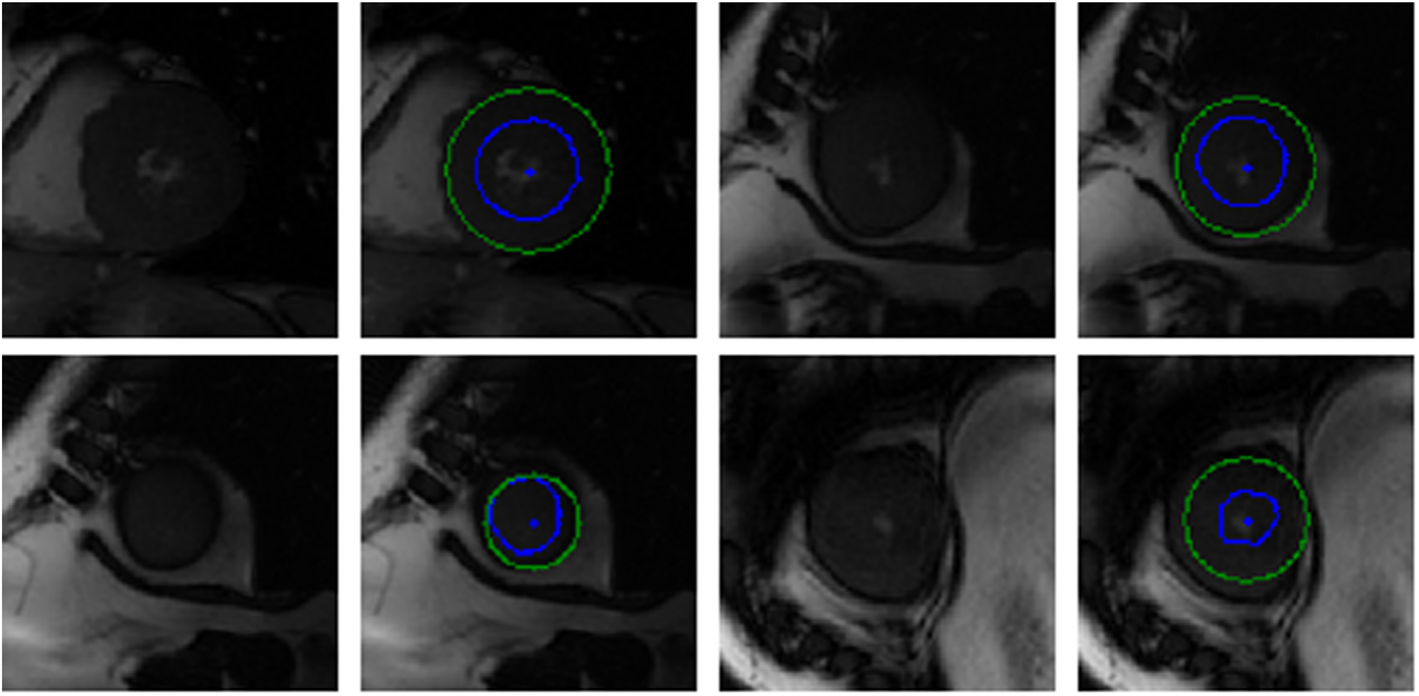
**Bad quality Sunnybrook automatic segmentations.** Selection of images from validation patient images with hypertrophy and their low quality automatically segmented contours (inner contour is blue and outer is green). The blue dot in the middle of the endocardium is the estimated centre point.

Our segmentation process performs well on those subjects in the evaluation set with normal heart function (SC-N) and those with heart failure with (SC-HF-I) and without infarction (SC-HF-NI). Our process performs worst on the inner contour of patients in the evaluation set with hypertrophy (SC-HYP) as is illustrated in Figure 28. This effect is also observed in other models by other authors [25].

## Discussion

In this article, the CRF parameters are estimated using a gradient-free search approach. The time spent searching for parameters can be further reduced by considering only a subset of the training videos. Due to the relatively low dimensionality of the parameter search space (≈14 dimensions) and relatively informative nature of each video sequence for all parameters, data scarcity is not a problem. It is, however, advisable that the training set contains a sufficient number of video sequences in which the papillary muscles obscure the endocardium border.

Note that no image preprocessing is done to compensate for effects such as different intensity settings on the MRI equipment. Image equalisation before segmentation would likely result in more robust result.

Inclusion of spatially neighbouring slices into a unified 3D and time model might also increase segmentation accuracy. Information at spatially higher slices, where the papillary muscles are less problematic, could then be used to improve the accuracy at lower slices. Linking the radial values between different slices would be relatively simple. This can be done with appropriate feature functions similar to those restricting radial continuity in a single contour. This would, however, require aligning slices to compensate for translation caused by different levels of inhalation and expiration between slices. It should also be simple to extend the segmentation process to include these additional features by adding them to the propagated messages.

Ideally, the time of end-systole in (14) should not be decided before inference, however, this might require a second order CRF or modelling as an additional unobserved variable. This would lead to an increase in segmentation time. Alternatively, techniques based on detecting temporary suspension and reversal of optical flow could be useful in detecting end-systole.

A second order system would also make the incorporation of contour smoothness information possible, as currently only contour continuity is taken into account. Alternatively, post-processing of the resultant contours by fitting them to splines, would improve smoothness.

From a visual inspection of the ground truth it is clear that there are inconsistencies in the human annotations with regard to the inclusion of the papillary muscles in the inner contour. These inconsistencies reduce the discriminative ability of the edge classifier and influences the optimal CRF parameter values estimated during training. Also, because the human annotated contours are used for evaluation, inconsistencies of the human annotations need to be taken into account when interpreting any results based on this as ground truth. In short: inconsistent examples in the training and evaluation set will result in an upper limit to the accuracy achievable by any consistent system.

## Conclusion

We present a CRF implementation for the automated segmentation of the left ventricle. Features are derived from discriminative properties of a human annotated dataset. The algorithm exhibits robustness against inclusion of the papillary muscles by integrating shape and motion information.

Experiments on the Sunnybrook dataset suggests that our technique would provide segmentations superior to those reported in the challenge.

The most significant segmentation errors are present in images of patients with hypertrophy, when the blood pool disappears from view. This limitation is due to the assumption that the inner contour is present in each frame. Future work could address these failures, possibly through the modelling of the right ventricle’s center point to avoid the LV outer contour from snapping to the outer edge of the entire heart structure.

Additional modelling of the right ventricle would also be beneficial, but is complicated by the polar coordinate space formulation, which allows only for one center point per frame.

Future work could also include faster and more robust centre point estimation. As mentioned previously, framing the centre point estimation in a model that can be solved with dynamic programming allows us to formulate its optimization as a belief propagation algorithm. This has the advantage that the centre point can be estimated as an additional latent variable in our CRF model, that needs to be inferred. Alternating between inferring ***ρ*** and ***c*** is thus theoretically possible, although insufficient research has been done as to its practical value.

## Competing interests

The authors declare they have no competing interests.

## Authors’ contributions

JD developed the model, wrote the software and wrote most of the paper. BH and JdP contributed to the analysis of the model and co-authored the paper. All authors read and approved the final manuscript.

## Pre-publication history

The pre-publication history for this paper can be accessed here:

http://www.biomedcentral.com/1471-2342/13/24/prepub

## Supplementary Material

Additional file 1 **Automatically segmented videos.** A video containing automatically annotated sequences from different patients for the York dataset. Click here for file

Additional file 2 **Large Sunnybrook montage image.** Montage of all end-systole and end-diastole automatic segmentations for the Sunnybrook dataset. Click here for file
