# Supplementary figures and images for: Left ventricular segmentation from MRI datasets with edge modelling conditional random fields
Source: BMC Med Imaging. 2013 Jul 31;13:24. doi: 10.1186/1471-2342-13-24 (PMC3737053; doi:10.1186/1471-2342-13-24)

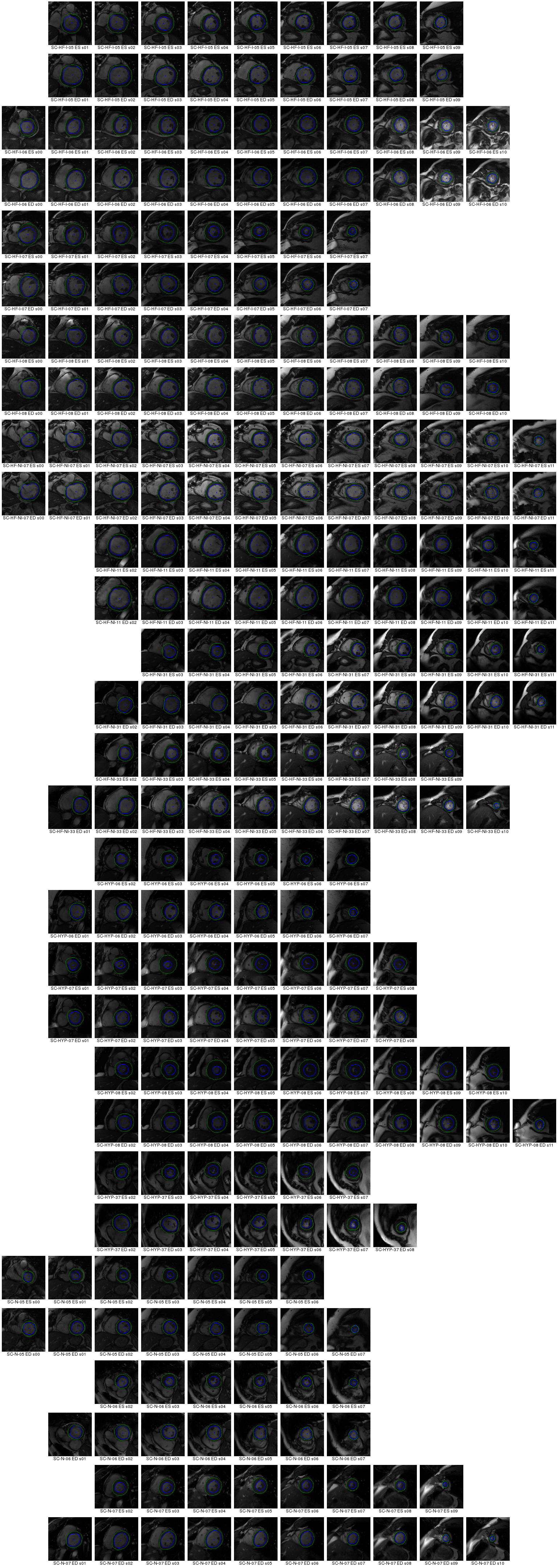

Supplement: Additional file 2 — Large Sunnybrook montage image. Montage of all end-systole and end-diastole automatic segmentations for the Sunnybrook dataset. [file 1471-2342-13-24-S2.png]
